# Supplementary material for: GD2 Expression in Medulloblastoma and Neuroblastoma for Personalized Immunotherapy: A Matter of Subtype
Source: Cancers (Basel). 2022 Dec 8;14(24):6051. doi: 10.3390/cancers14246051 (PMC9775636; doi:10.3390/cancers14246051)
Supplement: Supplementary file 1 [file cancers-14-06051-s001.zip › Supplemental_material_S1.html]

Medulloblastoma analysis


Code 

- Show All Code
- Hide All Code
- Download Rmd

# Medulloblastoma analysis

#### Arsenij Ustjanzew (arsenij.ustjanzew@uni-mainz.de) IMBEI, University Medical Center Mainz

#### 01.06.2022

# 1 Load necessary packages

```
library("SummarizedExperiment")
library("DT")
library("DESeq2")
library("org.Hs.eg.db")
library("pheatmap")
library("dplyr")
#devtools::install_github("vqv/ggbiplot")
library("ggbiplot")
library("KEGGREST")
library(viridis)
```

# 2 Read data

Data was used from Circular and Fusion RNAs in Medulloblastoma Development by Ani Azatyan andPeter G. Zaphiropoulos, 2022 (https://doi.org/10.3390/cancers14133134).

- Raw count matrix was downloaded from Gene Expression Omnibus with the GEO accession GSE203174.
- Sample metadata was extracted from the supplementary material Table S1 Medulloblastoma (MB) tumor and normal cerebellum (CB) sample information

```
counts <- read.table("./data/GSE203174/mRNA_raw_counts_matrix.txt", sep="\t", header=TRUE)
metadata <- read.table("./data/GSE203174/sample_information.txt", sep="\t", header=TRUE)
```

# 3 Modify data

Row data:
- Convert ensembl IDs into gene symbols and entrez IDs

Count matrix:
- Use column “id” for rownames and remove this column
- Keep only samples with “m\_” (RNA-Seq)
- Modify column names

Column data:
- create rownames identical to count matrix sample names
- keep neccessary columns
- merge subgroups “\_prog”, “\_rec” into the respective groups

- reorder samples of the count matrix based on column data

```
# row data
ensemblIDs <- gsub("\\..*", "", counts$id)
geneSymbol <- AnnotationDbi::mapIds(org.Hs.eg.db, keys = ensemblIDs, keytype = "ENSEMBL", column="SYMBOL")
```

```
## 'select()' returned 1:many mapping between keys and columns
```

```
entrezIDs <- AnnotationDbi::mapIds(org.Hs.eg.db, keys = ensemblIDs, keytype = "ENSEMBL", column="ENTREZID")
```

```
## 'select()' returned 1:many mapping between keys and columns
```

```
rowdata <- data.frame(ensemblIDs = ensemblIDs, geneSymbol = geneSymbol, entrezIDs = entrezIDs, row.names = counts$id)
# View(rowdata)

# count data
rownames(counts) <- counts[,1]
counts <- counts[,-which(colnames(counts)=="id")]
counts <- counts[,grepl("m_", colnames(counts))]
colnames(counts) <- gsub("_.*", "", colnames(counts))

# View(counts)

# column data
coldata_id  <- metadata$Label_RNA.seq.type..mRNA.
coldata_id[1:9] <- paste0("0", coldata_id[1:9])
coldata_id <- gsub("-", "", coldata_id)
coldata_id <- paste0("X", coldata_id)

all(coldata_id %in% colnames(counts))
```

```
## [1] TRUE
```

```
all(colnames(counts) %in% coldata_id)
```

```
## [1] TRUE
```

```
coldata <- metadata[,c("Label_RNA.seq.type..mRNA.","Group","Biospecimen.id","Research.subject.id","Location.and.additional.information")]
coldata$Groups <- coldata$Group
coldata$Groups <- gsub("_.*", "", coldata$Groups)

rownames(coldata) <- coldata_id

# View(coldata)

# reorder counts based on coldata
counts <- counts[,coldata_id]
```

# 4 Create DESeq2 object

```
dds <- DESeqDataSetFromMatrix(countData = counts,
                              colData = coldata,
                              rowData = rowdata,
                              design= ~ Groups)
```

```
## Warning in DESeqDataSet(se, design = design, ignoreRank): some variables in
## design formula are characters, converting to factors
```

```
dds
```

```
## class: DESeqDataSet 
## dim: 59586 86 
## metadata(1): version
## assays(1): counts
## rownames(59586): ENSG00000223972.5 ENSG00000227232.5 ... IGL.g@-ext
##   IGL-.g@-ext
## rowData names(3): ensemblIDs geneSymbol entrezIDs
## colnames(86): X01m X02m ... X85m X86m
## colData names(6): Label_RNA.seq.type..mRNA. Group ...
##   Location.and.additional.information Groups
```

```
#saveRDS(dds, "./data/GSE203174/GSE203174_dds.Rds")
```

## 4.1 Data overview:

Count data consists of 86 columns (samples) and 59586 rows (transcripts).

Nr. of samples per group:

```
table(as.factor(colData(dds)$Group))
```

```
## 
##          CB      Group3  Group3_rec      Group4 Group4_prog  Group4_rec 
##           5          15           2          32           1           2 
##         SHH    SHH_prog         WNT 
##          19           4           6
```

Nr. of samples per big group:

```
table(colData(dds)$Groups)
```

```
## 
##     CB Group3 Group4    SHH    WNT 
##      5     17     35     23      6
```

# 5 Minimal pre-filtering

Filtering genes with less then 10 counts in sum for all samples

```
keep <- rowSums(counts(dds)) >= 10
dds <- dds[keep,]
```

# 6 Normalization & log10 +1 & subset to genes of interest

- DESeq2 normalization
- log10 + 1 transformation
- Subsetting to genes of interest

Genes of interest are obtained from the four KEGG pathways:

- Sphingolipid metabolism (hsa00600)
- Glycosphingolipid biosynthesis - lacto and neolacto series (hsa00601)
- Glycosphingolipid biosynthesis - globo and isoglobo series (hsa00603)
- Glycosphingolipid biosynthesis - ganglio series (hsa00604)

```
dds <- estimateSizeFactors(dds)

target_genes <- readRDS("./data/target_genes.Rds")
norm_counts <- counts(dds[which(rowData(dds)$geneSymbol %in% c(target_genes$SYMBOL, "ST8SIA3")), ], normalized=TRUE)
norm_counts <- log10(norm_counts + 1)

rownames(norm_counts) <- rowData(dds)$geneSymbol[which(rowData(dds)$geneSymbol %in% c(target_genes$SYMBOL, "ST8SIA3"))]
```

# 7 Overview genes of interest

Heatmap showing the median log10 normalized gene expression per group.

```
ph_df <- t(norm_counts) %>% as.data.frame()
ph_df$group <- colData(dds)$Groups

ph_df %<>%
    group_by(group) %>%
    dplyr::summarise(across(everything(), median)) %>%
    as.data.frame()

rownames(ph_df) <- ph_df$group
ph_df <- ph_df[,-which(colnames(ph_df)=="group")]

pheatmap(t(ph_df), angle_col="45", cellheight=12)
```

# 8 PCA

Retrieving genes from KEGG pathways

```
gl_pw1 <- keggLink("hsa","hsa00600") # Sphingolipid metabolism
gl_pw2 <- keggLink("hsa","hsa00601") # Glycosphingolipid biosynthesis - lacto and neolacto series
gl_pw3 <- keggLink("hsa","hsa00603") # Glycosphingolipid biosynthesis - globo and isoglobo series
gl_pw4 <- keggLink("hsa","hsa00604") # Glycosphingolipid biosynthesis - ganglio series

ids_pw1 <- gsub("hsa:", "", gl_pw1)
pw1_symbolIDs <- AnnotationDbi::mapIds(org.Hs.eg.db, keys = ids_pw1, keytype = "ENTREZID", column="SYMBOL")
```

```
## 'select()' returned 1:1 mapping between keys and columns
```

```
#View(pw1_symbolIDs)

ids_pw2 <- gsub("hsa:", "", gl_pw2)
pw2_symbolIDs <- AnnotationDbi::mapIds(org.Hs.eg.db, keys = ids_pw2, keytype = "ENTREZID", column="SYMBOL")
```

```
## 'select()' returned 1:1 mapping between keys and columns
```

```
#View(pw2_symbolIDs)

ids_pw3 <- gsub("hsa:", "", gl_pw3)
pw3_symbolIDs <- AnnotationDbi::mapIds(org.Hs.eg.db, keys = ids_pw3, keytype = "ENTREZID", column="SYMBOL")
```

```
## 'select()' returned 1:1 mapping between keys and columns
```

```
#View(pw3_symbolIDs)

ids_pw4 <- gsub("hsa:", "", gl_pw4)
pw4_symbolIDs <- AnnotationDbi::mapIds(org.Hs.eg.db, keys = ids_pw4, keytype = "ENTREZID", column="SYMBOL")
```

```
## 'select()' returned 1:1 mapping between keys and columns
```

## 8.1 PCA based on the most important known enzymes in ganglioside biosynthesis: ST3GAL5, ST8SIA1, ST8SIA5, B3GALT4, B4GALNT1, and B4GALT6

```
pca <- prcomp(t(norm_counts[c("ST3GAL5", "ST8SIA1", "ST8SIA5", "B3GALT4", "B4GALNT1", "B4GALT6"),]))

ggbiplot(pca, choices = 1:2, obs.scale = 1, var.scale = 1,
         groups = colData(dds)$Groups, 
         ellipse = T, circle = F) +
  scale_color_discrete(name = '') +
  theme(legend.direction = 'horizontal', legend.position = 'top')
```

## 8.2 PCA based on genes of the Sphingolipid metabolism pathway.

```
pca <- prcomp(t(norm_counts[which(rownames(norm_counts) %in% pw1_symbolIDs),]))

ggbiplot(pca, choices = 1:2, obs.scale = 1, var.scale = 1,
         groups = colData(dds)$Groups, 
         ellipse = T, circle = F, var.axes=F) +
  scale_color_discrete(name = '') +
  theme(legend.direction = 'horizontal', legend.position = 'top')
```

## 8.3 PCA based on genes of the Glycosphingolipid biosynthesis - lacto and neolacto series pathway.

```
pca <- prcomp(t(norm_counts[which(rownames(norm_counts) %in% pw2_symbolIDs),]))

ggbiplot(pca, choices = 1:2, obs.scale = 1, var.scale = 1,
         groups = colData(dds)$Groups, 
         ellipse = T, circle = F, var.axes=F) +
  scale_color_discrete(name = '') +
  theme(legend.direction = 'horizontal', legend.position = 'top')
```

## 8.4 PCA based on genes of the Glycosphingolipid biosynthesis - globo and isoglobo series pathway.

```
pca <- prcomp(t(norm_counts[which(rownames(norm_counts) %in% pw3_symbolIDs),]))

ggbiplot(pca, choices = 1:2, obs.scale = 1, var.scale = 1,
         groups = colData(dds)$Groups, 
         ellipse = T, circle = F, var.axes=F) +
  scale_color_discrete(name = '') +
  theme(legend.direction = 'horizontal', legend.position = 'top')
```

## 8.5 PCA based on genes of the Glycosphingolipid biosynthesis - ganglio series pathway.

```
pca <- prcomp(t(norm_counts[which(rownames(norm_counts) %in% pw4_symbolIDs),]))

ggbiplot(pca, choices = 1:2, obs.scale = 1, var.scale = 1,
         groups = colData(dds)$Groups, 
         ellipse = T, circle = F, var.axes=F) +
  scale_color_discrete(name = '') +
  theme(legend.direction = 'horizontal', legend.position = 'top')
```

## 8.6 PCA based on all genes of the four KEGG pathways

```
# pca <- prcomp(t(norm_counts))
# 
# dtp <- data.frame('Groups' = colData(dds)$Groups, pca$x[,1:3])
# plotly::plot_ly(dtp, x = ~PC1, y = ~PC2, z = ~PC3, color = ~Groups)

pca <- prcomp(t(norm_counts))

ggbiplot(pca, choices = 1:2, obs.scale = 1, var.scale = 1,
         groups = colData(dds)$Groups, 
         ellipse = T, circle = F, var.axes=F) +
  scale_color_discrete(name = '') +
  theme(legend.direction = 'horizontal', legend.position = 'top')
```

# 9 Differential Gene Expression Analysis

```
FDR <- 0.05

dds <- dds[!duplicated(rowData(dds)$ensemblIDs),]
rownames(dds) <- rowData(dds)$ensemblIDs
# ideal::ideal(dds)

colData(dds)$Groups <- relevel(colData(dds)$Groups, "SHH")

dds <- DESeq2::DESeq(dds, parallel = TRUE)
```

```
## using pre-existing size factors
```

```
## estimating dispersions
```

```
## gene-wise dispersion estimates: 30 workers
```

```
## mean-dispersion relationship
```

```
## final dispersion estimates, fitting model and testing: 30 workers
```

```
## -- replacing outliers and refitting for 1791 genes
## -- DESeq argument 'minReplicatesForReplace' = 7 
## -- original counts are preserved in counts(dds)
```

```
## estimating dispersions
```

```
## fitting model and testing
```

```
resultsNames(dds)
```

```
## [1] "Intercept"            "Groups_CB_vs_SHH"     "Groups_Group3_vs_SHH"
## [4] "Groups_Group4_vs_SHH" "Groups_WNT_vs_SHH"
```

```
summary(results(dds))
```

```
## 
## out of 39584 with nonzero total read count
## adjusted p-value < 0.1
## LFC > 0 (up)       : 3886, 9.8%
## LFC < 0 (down)     : 5603, 14%
## outliers [1]       : 102, 0.26%
## low counts [2]     : 9981, 25%
## (mean count < 1)
## [1] see 'cooksCutoff' argument of ?results
## [2] see 'independentFiltering' argument of ?results
```

## 9.1 CB vs SHH

```
dds_res1 <- results(dds, alpha=FDR, contrast=c("Groups", "CB", "SHH"))

summary(dds_res1)
```

```
## 
## out of 39584 with nonzero total read count
## adjusted p-value < 0.05
## LFC > 0 (up)       : 6085, 15%
## LFC < 0 (down)     : 6418, 16%
## outliers [1]       : 102, 0.26%
## low counts [2]     : 8458, 21%
## (mean count < 1)
## [1] see 'cooksCutoff' argument of ?results
## [2] see 'independentFiltering' argument of ?results
```

```
dds_res1_df <- as.data.frame(dds_res1)

dds_res1_df <- dds_res1_df[rownames(dds_res1_df) %in% target_genes$ENSEMBL,]

dds_res1_df <- round(dds_res1_df, 5)

dds_res1_df$GeneSymbol <- AnnotationDbi::mapIds(org.Hs.eg.db, keys = rownames(dds_res1_df), keytype = "ENSEMBL", column="SYMBOL")
```

```
## 'select()' returned 1:1 mapping between keys and columns
```

```
DT::datatable(dds_res1_df, caption="Groups_CB_vs_SHH, DE genes (rounded values)")
```

## 9.2 Group3 vs SHH

```
dds_res2 <- results(dds, alpha=FDR, contrast=c("Groups", "Group3", "SHH"))

summary(dds_res2)
```

```
## 
## out of 39584 with nonzero total read count
## adjusted p-value < 0.05
## LFC > 0 (up)       : 6901, 17%
## LFC < 0 (down)     : 5709, 14%
## outliers [1]       : 102, 0.26%
## low counts [2]     : 7691, 19%
## (mean count < 1)
## [1] see 'cooksCutoff' argument of ?results
## [2] see 'independentFiltering' argument of ?results
```

```
dds_res2_df <- as.data.frame(dds_res2)

dds_res2_df <- dds_res2_df[rownames(dds_res2_df) %in% target_genes$ENSEMBL,]

dds_res2_df <- round(dds_res2_df, 5)

dds_res2_df$GeneSymbol <- AnnotationDbi::mapIds(org.Hs.eg.db, keys = rownames(dds_res2_df), keytype = "ENSEMBL", column="SYMBOL")
```

```
## 'select()' returned 1:1 mapping between keys and columns
```

```
DT::datatable(dds_res2_df, caption="Groups_Group3_vs_SHH, DE genes (rounded values)")
```

## 9.3 Group4 vs SHH

```
dds_res3 <- results(dds, alpha=FDR, contrast=c("Groups", "Group4", "SHH"))

summary(dds_res3)
```

```
## 
## out of 39584 with nonzero total read count
## adjusted p-value < 0.05
## LFC > 0 (up)       : 7613, 19%
## LFC < 0 (down)     : 7194, 18%
## outliers [1]       : 102, 0.26%
## low counts [2]     : 6925, 17%
## (mean count < 0)
## [1] see 'cooksCutoff' argument of ?results
## [2] see 'independentFiltering' argument of ?results
```

```
dds_res3_df <- as.data.frame(dds_res3)

dds_res3_df <- dds_res3_df[rownames(dds_res3_df) %in% target_genes$ENSEMBL,]

dds_res3_df <- round(dds_res3_df, 5)

dds_res3_df$GeneSymbol <- AnnotationDbi::mapIds(org.Hs.eg.db, keys = rownames(dds_res3_df), keytype = "ENSEMBL", column="SYMBOL")
```

```
## 'select()' returned 1:1 mapping between keys and columns
```

```
DT::datatable(dds_res3_df, caption="Groups_Group4_vs_SHH, DE genes (rounded values)")
```

## 9.4 WNT vs SHH

```
dds_res4 <- results(dds, alpha=FDR, contrast=c("Groups", "WNT", "SHH"))

summary(dds_res4)
```

```
## 
## out of 39584 with nonzero total read count
## adjusted p-value < 0.05
## LFC > 0 (up)       : 3146, 7.9%
## LFC < 0 (down)     : 4465, 11%
## outliers [1]       : 102, 0.26%
## low counts [2]     : 9219, 23%
## (mean count < 1)
## [1] see 'cooksCutoff' argument of ?results
## [2] see 'independentFiltering' argument of ?results
```

```
dds_res4_df <- as.data.frame(dds_res4)

dds_res4_df <- dds_res4_df[rownames(dds_res4_df) %in% target_genes$ENSEMBL,]

dds_res4_df <- round(dds_res4_df, 5)

dds_res4_df$GeneSymbol <- AnnotationDbi::mapIds(org.Hs.eg.db, keys = rownames(dds_res4_df), keytype = "ENSEMBL", column="SYMBOL")
```

```
## 'select()' returned 1:1 mapping between keys and columns
```

```
DT::datatable(dds_res4_df, caption="Groups_WNT_vs_SHH, DE genes (rounded values)")
```

Generating list with all genes where P adjusted value < 0.05 in 3 or more comparisons

```
genelist <-c(
  dds_res1_df[dds_res1_df$padj<=0.05,"GeneSymbol"],
  dds_res2_df[dds_res2_df$padj<=0.05,"GeneSymbol"],
  dds_res3_df[dds_res3_df$padj<=0.05,"GeneSymbol"],
  dds_res4_df[dds_res4_df$padj<=0.05,"GeneSymbol"]
)

genelist_freq <- table(genelist[!is.na(genelist)])
genelist_freq <- genelist_freq[genelist_freq>2]
genelist <- names(genelist_freq)
genelist
```

```
##  [1] "A4GALT"     "ABO"        "ACER3"      "ARSA"       "B3GALT2"   
##  [6] "B3GNT2"     "B3GNT5"     "B4GALT2"    "B4GALT3"    "B4GALT5"   
## [11] "B4GALT6"    "CERS4"      "FUT1"       "FUT2"       "FUT3"      
## [16] "FUT4"       "FUT6"       "FUT7"       "GBA"        "GBA2"      
## [21] "GCNT2"      "GLA"        "NEU1"       "NEU4"       "SGPP2"     
## [26] "SLC33A1"    "SPHK1"      "ST3GAL6"    "ST6GALNAC6" "ST8SIA1"   
## [31] "ST8SIA5"
```

```
f <- rep(seq_len(ceiling(length(genelist) / 2)),each = 2,length.out = length(genelist))

for(i in unique(f)){
  p <- caret::featurePlot(x = t(norm_counts)[,genelist[which(f %in% i)]],
            y = colData(dds)$Groups,
            plot = "box",
            scales = list(x = list(relation="free", rot=90),
                          y = list(relation="free")))
  print(p)
}
```

# 10 Two gene signature ST8SIA1 & B4GALNT

```
sig_df <- t(norm_counts[c("ST8SIA1", "B4GALNT1"),]) %>% as.data.frame()
sig_df$ST8SIA1_B4GALNT1 <- sig_df$ST8SIA1 + sig_df$B4GALNT1
sig_df$Groups <- as.factor(colData(dds)$Groups)
sig_df$Sample <- rownames(sig_df)

# sig_df_long <- sig_df %>%
#       group_by(Groups) %>%
#       tidyr::pivot_longer(colnames(dplyr::select(sig_df, !c("Sample", "Groups"))), names_to = "gene", values_to = "value")

p <- ggplot(sig_df, aes(x = Groups, y = ST8SIA1_B4GALNT1, fill = Groups)) +
  geom_boxplot() +
  scale_fill_viridis(discrete = TRUE, alpha = 0.6) +
  geom_jitter(color = "black",
              size = 0.4,
              alpha = 0.9) +
  theme_classic() +
  theme(legend.position = "none",
        plot.title = element_text(size = 11)) +
  ggtitle("Two gene signature") +
  ylab("log10( ST8SIA1 + B4GALNT1 )") +
  xlab("")
p
```

## 10.1 Two gene signature with Neuroblastoma

```
nbSE <- readRDS("./data/xenabrowser/NB_gene_expected_count.Rds")

row.names(nbSE) <- gsub("\\..*", "", rownames(nbSE))
assays(nbSE)$integerCounts <- round(((2^assays(nbSE)$counts) - 1), 0)

common_genes <- intersect(rownames(dds), rownames(nbSE))
counts_dds <- counts(dds[common_genes,], normalized=FALSE)
counts_nb <- assays(nbSE)$integerCounts[common_genes,]
counts_dds_nb <- cbind(counts_dds, counts_nb)


coldata_dds_nb <- data.frame(Group=c(colData(dds)$Groups, as.factor(colData(nbSE)$detailed_category)))

levels(coldata_dds_nb$Group) <- c("SHH","CB","Group3","Group4","WNT","NBL")

dds_nbl <- DESeq2::DESeqDataSetFromMatrix(counts_dds_nb, coldata_dds_nb, design=~Group)
```

```
## converting counts to integer mode
```

```
dds_nbl <- estimateSizeFactors(dds_nbl)
norm_counts <- counts(dds_nbl[which(rownames(dds_nbl) %in% c(target_genes$ENSEMBL)), ], normalized=TRUE)
norm_counts <- log10(norm_counts + 1)

rownames(norm_counts) <- AnnotationDbi::mapIds(org.Hs.eg.db, keys = rownames(norm_counts), keytype = "ENSEMBL", column="SYMBOL")
```

```
## 'select()' returned 1:1 mapping between keys and columns
```

```
sig_df <- t(norm_counts[c("ST8SIA1", "B4GALNT1"),]) %>% as.data.frame()
sig_df$ST8SIA1_B4GALNT1 <- sig_df$ST8SIA1 + sig_df$B4GALNT1
sig_df$Group <- as.factor(colData(dds_nbl)$Group)
sig_df$Sample <- rownames(sig_df)

# sig_df_long <- sig_df %>%
#       group_by(Groups) %>%
#       tidyr::pivot_longer(colnames(dplyr::select(sig_df, !c("Sample", "Groups"))), names_to = "gene", values_to = "value")

p <- ggplot(sig_df, aes(x = Group, y = ST8SIA1_B4GALNT1, fill = Group)) +
  geom_boxplot() +
  scale_fill_viridis(discrete = TRUE, alpha = 0.6) +
  geom_jitter(color = "black",
              size = 0.4,
              alpha = 0.9) +
  theme_classic() +
  theme(legend.position = "none",
        plot.title = element_text(size = 11)) +
  ggtitle("Two gene signature") +
  ylab("log10( ST8SIA1 + B4GALNT1 )") +
  xlab("")
p
```

```
rm(nbSE)
rm(counts_dds)
rm(counts_nb)
rm(dds_nbl)
rm(counts_dds_nb)
gc()
```

```
##            used  (Mb) gc trigger  (Mb)  max used  (Mb)
## Ncells  7697336 411.1   12142307 648.5  12142307 648.5
## Vcells 33279978 254.0  106529360 812.8 106528577 812.8
```

# 11 Two gene signature of MB combined with other data sets

## 11.1 GTEx + MB

```
gtexSE <- readRDS("./data/xenabrowser/GTEx_gene_expected_count.Rds")

row.names(gtexSE) <- gsub("\\..*", "", rownames(gtexSE))
assays(gtexSE)$integerCounts <- round(((2^assays(gtexSE)$counts) - 1), 0)

common_genes <- intersect(rownames(dds), rownames(gtexSE))
counts_dds <- counts(dds[common_genes,], normalized=FALSE)
counts_gtex <- assays(gtexSE)$integerCounts[common_genes,]
counts_dds_gtex <- cbind(counts_dds, counts_gtex)

colData(gtexSE)$category_extended <- paste0(colData(gtexSE)$detailed_category, " (", colData(gtexSE)$X_sample_type, ")")
coldata_dds_gtex <- data.frame(Group=c(colData(dds)$Groups, as.factor(colData(gtexSE)$category_extended)))

#levels(coldata_dds_nb$Group) <- c("SHH","CB","Group3","Group4","WNT","NBL")

dds_gtex <- DESeq2::DESeqDataSetFromMatrix(counts_dds_gtex, coldata_dds_gtex, design=~Group)
```

```
## converting counts to integer mode
```

```
##   Note: levels of factors in the design contain characters other than
##   letters, numbers, '_' and '.'. It is recommended (but not required) to use
##   only letters, numbers, and delimiters '_' or '.', as these are safe characters
##   for column names in R. [This is a message, not a warning or an error]
```

```
dds_gtex <- estimateSizeFactors(dds_gtex)
```

```
##   Note: levels of factors in the design contain characters other than
##   letters, numbers, '_' and '.'. It is recommended (but not required) to use
##   only letters, numbers, and delimiters '_' or '.', as these are safe characters
##   for column names in R. [This is a message, not a warning or an error]
```

```
norm_counts <- counts(dds_gtex[which(rownames(dds_gtex) %in% c(target_genes$ENSEMBL)), ], normalized=TRUE)
norm_counts <- log10(norm_counts + 1)

rownames(norm_counts) <- AnnotationDbi::mapIds(org.Hs.eg.db, keys = rownames(norm_counts), keytype = "ENSEMBL", column="SYMBOL")
```

```
## 'select()' returned 1:1 mapping between keys and columns
```

```
norm_counts_sub <- t(norm_counts[c("ST3GAL5", "ST8SIA1", "ST8SIA5", "B3GALT4", "B4GALNT1", "B4GALT6"),]) %>% as.data.frame()
norm_counts_sub$ST8SIA1_B4GALNT1 <- norm_counts_sub$ST8SIA1 + norm_counts_sub$B4GALNT1
norm_counts_sub$Group <- colData(dds_gtex)$Group

norm_counts_sub %<>%
    group_by(Group) %>%
    dplyr::summarise(across(everything(), median)) %>%
    as.data.frame()

rownames(norm_counts_sub) <- norm_counts_sub$Group
norm_counts_sub <- norm_counts_sub[,-which(colnames(norm_counts_sub)=="Group")]

norm_counts_sub <- norm_counts_sub[order(norm_counts_sub$ST8SIA1_B4GALNT1, decreasing = TRUE),]

pheatmap(norm_counts_sub, angle_col="45", cellheight=12, cluster_rows = FALSE, cluster_cols = FALSE)
```

```
rm(gtexSE)
rm(counts_dds)
rm(counts_gtex)
rm(counts_dds_gtex)
rm(norm_counts)
rm(norm_counts_sub)
rm(dds_gtex)
gc()
```

```
##            used  (Mb) gc trigger    (Mb)   max used    (Mb)
## Ncells  7721113 412.4   12142307   648.5   12142307   648.5
## Vcells 33293157 254.1 2064819201 15753.4 2581006152 19691.6
```

## 11.2 TARGET + MB

```
targetSE <- readRDS("./data/xenabrowser/TARGET_gene_expected_count.Rds")

row.names(targetSE) <- gsub("\\..*", "", rownames(targetSE))
assays(targetSE)$integerCounts <- round(((2^assays(targetSE)$counts) - 1), 0)

common_genes <- intersect(rownames(dds), rownames(targetSE))
counts_dds <- counts(dds[common_genes,], normalized=FALSE)
counts_target <- assays(targetSE)$integerCounts[common_genes,]
counts_dds_target <- cbind(counts_dds, counts_target)

colData(targetSE)$category_extended <- paste0(colData(targetSE)$detailed_category, " (", colData(targetSE)$X_sample_type, ")")
coldata_dds_target <- data.frame(Group=c(colData(dds)$Groups, as.factor(colData(targetSE)$category_extended)))

#levels(coldata_dds_nb$Group) <- c("SHH","CB","Group3","Group4","WNT","NBL")

dds_target <- DESeq2::DESeqDataSetFromMatrix(counts_dds_target, coldata_dds_target, design=~Group)
```

```
## converting counts to integer mode
```

```
##   Note: levels of factors in the design contain characters other than
##   letters, numbers, '_' and '.'. It is recommended (but not required) to use
##   only letters, numbers, and delimiters '_' or '.', as these are safe characters
##   for column names in R. [This is a message, not a warning or an error]
```

```
dds_target <- estimateSizeFactors(dds_target)
```

```
##   Note: levels of factors in the design contain characters other than
##   letters, numbers, '_' and '.'. It is recommended (but not required) to use
##   only letters, numbers, and delimiters '_' or '.', as these are safe characters
##   for column names in R. [This is a message, not a warning or an error]
```

```
norm_counts <- counts(dds_target[which(rownames(dds_target) %in% c(target_genes$ENSEMBL)), ], normalized=TRUE)
norm_counts <- log10(norm_counts + 1)

rownames(norm_counts) <- AnnotationDbi::mapIds(org.Hs.eg.db, keys = rownames(norm_counts), keytype = "ENSEMBL", column="SYMBOL")
```

```
## 'select()' returned 1:1 mapping between keys and columns
```

```
norm_counts_sub <- t(norm_counts[c("ST3GAL5", "ST8SIA1", "ST8SIA5", "B3GALT4", "B4GALNT1", "B4GALT6"),]) %>% as.data.frame()
norm_counts_sub$ST8SIA1_B4GALNT1 <- norm_counts_sub$ST8SIA1 + norm_counts_sub$B4GALNT1
norm_counts_sub$Group <- colData(dds_target)$Group

norm_counts_sub %<>%
    group_by(Group) %>%
    dplyr::summarise(across(everything(), median)) %>%
    as.data.frame()

rownames(norm_counts_sub) <- norm_counts_sub$Group
norm_counts_sub <- norm_counts_sub[,-which(colnames(norm_counts_sub)=="Group")]

norm_counts_sub <- norm_counts_sub[order(norm_counts_sub$ST8SIA1_B4GALNT1, decreasing = TRUE),]

pheatmap(norm_counts_sub, angle_col="45", cellheight=12, cluster_rows = FALSE, cluster_cols = FALSE)
```

```
rm(targetSE)
rm(counts_dds)
rm(counts_target)
rm(counts_dds_target)
rm(norm_counts)
rm(norm_counts_sub)
rm(dds_target)
gc()
```

```
##            used  (Mb) gc trigger    (Mb)   max used    (Mb)
## Ncells  7722889 412.5   12142307   648.5   12142307   648.5
## Vcells 33296621 254.1 1651855361 12602.7 2581006152 19691.6
```

## 11.3 TCGA + MB

```
tcgaSE <- readRDS("./data/xenabrowser/TCGA_gene_expected_count.Rds")

row.names(tcgaSE) <- gsub("\\..*", "", rownames(tcgaSE))
assays(tcgaSE)$integerCounts <- round(((2^assays(tcgaSE)$counts) - 1), 0)

common_genes <- intersect(rownames(dds), rownames(tcgaSE))
counts_dds <- counts(dds[common_genes,], normalized=FALSE)
counts_tcga <- assays(tcgaSE)$integerCounts[common_genes,]
counts_dds_tcga <- cbind(counts_dds, counts_tcga)

colData(tcgaSE)$category_extended <- paste0(colData(tcgaSE)$detailed_category, " (", colData(tcgaSE)$X_sample_type, ")")
coldata_dds_tcga <- data.frame(Group=c(colData(dds)$Groups, as.factor(colData(tcgaSE)$category_extended)))

#levels(coldata_dds_nb$Group) <- c("SHH","CB","Group3","Group4","WNT","NBL")

dds_tcga <- DESeq2::DESeqDataSetFromMatrix(counts_dds_tcga, coldata_dds_tcga, design=~Group)
```

```
## converting counts to integer mode
```

```
##   Note: levels of factors in the design contain characters other than
##   letters, numbers, '_' and '.'. It is recommended (but not required) to use
##   only letters, numbers, and delimiters '_' or '.', as these are safe characters
##   for column names in R. [This is a message, not a warning or an error]
```

```
dds_tcga <- estimateSizeFactors(dds_tcga)
```

```
##   Note: levels of factors in the design contain characters other than
##   letters, numbers, '_' and '.'. It is recommended (but not required) to use
##   only letters, numbers, and delimiters '_' or '.', as these are safe characters
##   for column names in R. [This is a message, not a warning or an error]
```

```
norm_counts <- counts(dds_tcga[which(rownames(dds_tcga) %in% c(target_genes$ENSEMBL)), ], normalized=TRUE)
norm_counts <- log10(norm_counts + 1)

rownames(norm_counts) <- AnnotationDbi::mapIds(org.Hs.eg.db, keys = rownames(norm_counts), keytype = "ENSEMBL", column="SYMBOL")
```

```
## 'select()' returned 1:1 mapping between keys and columns
```

```
norm_counts_sub <- t(norm_counts[c("ST3GAL5", "ST8SIA1", "ST8SIA5", "B3GALT4", "B4GALNT1", "B4GALT6"),]) %>% as.data.frame()
norm_counts_sub$ST8SIA1_B4GALNT1 <- norm_counts_sub$ST8SIA1 + norm_counts_sub$B4GALNT1
norm_counts_sub$Group <- colData(dds_tcga)$Group

norm_counts_sub %<>%
    group_by(Group) %>%
    dplyr::summarise(across(everything(), median)) %>%
    as.data.frame()

rownames(norm_counts_sub) <- norm_counts_sub$Group
norm_counts_sub <- norm_counts_sub[,-which(colnames(norm_counts_sub)=="Group")]

norm_counts_sub <- norm_counts_sub[order(norm_counts_sub$ST8SIA1_B4GALNT1, decreasing = TRUE),]

pheatmap(norm_counts_sub, angle_col="45", cellheight=12, cluster_rows = FALSE, cluster_cols = FALSE)
```

```
rm(tcgaSE)
rm(counts_dds)
rm(counts_tcga)
rm(counts_dds_tcga)
rm(norm_counts)
rm(norm_counts_sub)
rm(dds_tcga)
gc()
```

```
##            used  (Mb) gc trigger    (Mb)   max used    (Mb)
## Ncells  7754600 414.2   12142307   648.5   12142307   648.5
## Vcells 33324164 254.3 2743412226 20930.6 3429253247 26163.2
```

# 12 Session info

```
sessionInfo()
```

```
## R version 4.1.3 (2022-03-10)
## Platform: x86_64-pc-linux-gnu (64-bit)
## Running under: Ubuntu 20.04.4 LTS
## 
## Matrix products: default
## BLAS:   /usr/lib/x86_64-linux-gnu/openblas-pthread/libblas.so.3
## LAPACK: /usr/lib/x86_64-linux-gnu/openblas-pthread/liblapack.so.3
## 
## locale:
##  [1] LC_CTYPE=en_US.UTF-8       LC_NUMERIC=C              
##  [3] LC_TIME=en_US.UTF-8        LC_COLLATE=en_US.UTF-8    
##  [5] LC_MONETARY=en_US.UTF-8    LC_MESSAGES=en_US.UTF-8   
##  [7] LC_PAPER=en_US.UTF-8       LC_NAME=C                 
##  [9] LC_ADDRESS=C               LC_TELEPHONE=C            
## [11] LC_MEASUREMENT=en_US.UTF-8 LC_IDENTIFICATION=C       
## 
## attached base packages:
## [1] grid      stats4    stats     graphics  grDevices utils     datasets 
## [8] methods   base     
## 
## other attached packages:
##  [1] viridis_0.6.2               viridisLite_0.4.0          
##  [3] KEGGREST_1.34.0             ggbiplot_0.55              
##  [5] scales_1.2.0                plyr_1.8.7                 
##  [7] ggplot2_3.3.6               dplyr_1.0.9                
##  [9] pheatmap_1.0.12             org.Hs.eg.db_3.14.0        
## [11] AnnotationDbi_1.56.2        DESeq2_1.34.0              
## [13] DT_0.24                     SummarizedExperiment_1.24.0
## [15] Biobase_2.54.0              GenomicRanges_1.46.1       
## [17] GenomeInfoDb_1.30.1         IRanges_2.28.0             
## [19] S4Vectors_0.32.4            BiocGenerics_0.40.0        
## [21] MatrixGenerics_1.6.0        matrixStats_0.62.0         
## 
## loaded via a namespace (and not attached):
##   [1] colorspace_2.0-3       ellipsis_0.3.2         class_7.3-20          
##   [4] XVector_0.34.0         rstudioapi_0.13        listenv_0.8.0         
##   [7] farver_2.1.1           bit64_4.0.5            lubridate_1.8.0       
##  [10] prodlim_2019.11.13     fansi_1.0.3            codetools_0.2-18      
##  [13] splines_4.1.3          cachem_1.0.6           geneplotter_1.72.0    
##  [16] knitr_1.39             jsonlite_1.8.0         pROC_1.18.0           
##  [19] caret_6.0-93           annotate_1.72.0        png_0.1-7             
##  [22] compiler_4.1.3         httr_1.4.4             assertthat_0.2.1      
##  [25] Matrix_1.4-0           fastmap_1.1.0          cli_3.3.0             
##  [28] htmltools_0.5.3        tools_4.1.3            gtable_0.3.0          
##  [31] glue_1.6.2             GenomeInfoDbData_1.2.7 reshape2_1.4.4        
##  [34] Rcpp_1.0.9             jquerylib_0.1.4        vctrs_0.4.1           
##  [37] Biostrings_2.62.0      nlme_3.1-155           iterators_1.0.14      
##  [40] crosstalk_1.2.0        timeDate_4021.104      xfun_0.32             
##  [43] gower_1.0.0            stringr_1.4.0          globals_0.16.0        
##  [46] lifecycle_1.0.1        XML_3.99-0.10          future_1.27.0         
##  [49] zlibbioc_1.40.0        MASS_7.3-55            ipred_0.9-13          
##  [52] parallel_4.1.3         RColorBrewer_1.1-3     yaml_2.3.5            
##  [55] curl_4.3.2             memoise_2.0.1          gridExtra_2.3         
##  [58] sass_0.4.2             rpart_4.1.16           stringi_1.7.8         
##  [61] RSQLite_2.2.16         highr_0.9              genefilter_1.76.0     
##  [64] foreach_1.5.2          hardhat_1.2.0          BiocParallel_1.28.3   
##  [67] lava_1.6.10            rlang_1.0.4            pkgconfig_2.0.3       
##  [70] bitops_1.0-7           evaluate_0.16          lattice_0.20-45       
##  [73] purrr_0.3.4            recipes_1.0.1          htmlwidgets_1.5.4     
##  [76] labeling_0.4.2         bit_4.0.4              tidyselect_1.1.2      
##  [79] parallelly_1.32.1      magrittr_2.0.3         bookdown_0.28         
##  [82] R6_2.5.1               generics_0.1.3         DelayedArray_0.20.0   
##  [85] DBI_1.1.3              pillar_1.8.0           withr_2.5.0           
##  [88] nnet_7.3-17            survival_3.2-13        RCurl_1.98-1.8        
##  [91] future.apply_1.9.0     tibble_3.1.8           crayon_1.5.1          
##  [94] utf8_1.2.2             rmarkdown_2.15         locfit_1.5-9.6        
##  [97] data.table_1.14.2      blob_1.2.3             ModelMetrics_1.2.2.2  
## [100] digest_0.6.29          xtable_1.8-4           munsell_0.5.0         
## [103] bslib_0.4.0
```

LS0tCnRpdGxlOiAiTWVkdWxsb2JsYXN0b21hIGFuYWx5c2lzIgphdXRob3I6Ci0gbmFtZTogQXJzZW5paiBVc3RqYW56ZXcgKGFyc2VuaWoudXN0amFuemV3QHVuaS1tYWluei5kZSk8YnI+PGEgaHJlZj0naHR0cHM6Ly93d3cudW5pbWVkaXppbi1tYWluei5kZS9pbWJlaS8nPklNQkVJLAogICAgVW5pdmVyc2l0eSBNZWRpY2FsIENlbnRlciBNYWluejwvYT48YnI+CmRhdGU6ICIwMS4wNi4yMDIyIgpvdXRwdXQ6IAogIGJvb2tkb3duOjpodG1sX2RvY3VtZW50MjoKICAgIHRvYzogdHJ1ZQogICAgdG9jX2Zsb2F0OiB0cnVlCiAgICB0aGVtZTogY29zbW8KICAgIGNvZGVfZm9sZGluZzogc2hvdwogICAgY29kZV9kb3dubG9hZDogdHJ1ZQplZGl0b3Jfb3B0aW9uczoKICBjaHVua19vdXRwdXRfdHlwZTogY29uc29sZQotLS0KCmBgYHtyIHNldHVwLCBpbmNsdWRlPUZBTFNFfQprbml0cjo6b3B0c19jaHVuayRzZXQoZWNobyA9IFRSVUUpCmBgYAoKIyBMb2FkIG5lY2Vzc2FyeSBwYWNrYWdlcwoKYGBge3IgZWNobz1UUlVFLCB3YXJuaW5nPUZBTFNFLCBtZXNzYWdlPUZBTFNFLCByZXN1bHRzPSdoaWRlJ30KbGlicmFyeSgiU3VtbWFyaXplZEV4cGVyaW1lbnQiKQpsaWJyYXJ5KCJEVCIpCmxpYnJhcnkoIkRFU2VxMiIpCmxpYnJhcnkoIm9yZy5Icy5lZy5kYiIpCmxpYnJhcnkoInBoZWF0bWFwIikKbGlicmFyeSgiZHBseXIiKQojZGV2dG9vbHM6Omluc3RhbGxfZ2l0aHViKCJ2cXYvZ2diaXBsb3QiKQpsaWJyYXJ5KCJnZ2JpcGxvdCIpCmxpYnJhcnkoIktFR0dSRVNUIikKbGlicmFyeSh2aXJpZGlzKQpgYGAKCiMgUmVhZCBkYXRhCgpEYXRhIHdhcyB1c2VkIGZyb20gQ2lyY3VsYXIgYW5kIFtGdXNpb24gUk5BcyBpbiBNZWR1bGxvYmxhc3RvbWEgRGV2ZWxvcG1lbnQgYnkgQW5pIEF6YXR5YW4gYW5kUGV0ZXIgRy4gWmFwaGlyb3BvdWxvcywgMjAyMl0oaHR0cHM6Ly93d3cubWRwaS5jb20vMjA3Mi02Njk0LzE0LzEzLzMxMzQvaHRtKSAoaHR0cHM6Ly9kb2kub3JnLzEwLjMzOTAvY2FuY2VyczE0MTMzMTM0KS4KCi0gUmF3IGNvdW50IG1hdHJpeCB3YXMgZG93bmxvYWRlZCBmcm9tIFtHZW5lIEV4cHJlc3Npb24gT21uaWJ1c10oaHR0cHM6Ly93d3cubmNiaS5ubG0ubmloLmdvdi9nZW8vcXVlcnkvYWNjLmNnaT9hY2M9R1NFMjAzMTc0KSB3aXRoIHRoZSBHRU8gYWNjZXNzaW9uIEdTRTIwMzE3NC4KLSBTYW1wbGUgbWV0YWRhdGEgd2FzIGV4dHJhY3RlZCBmcm9tIHRoZSBzdXBwbGVtZW50YXJ5IG1hdGVyaWFsIFRhYmxlIFMxIE1lZHVsbG9ibGFzdG9tYSAoTUIpIHR1bW9yIGFuZCBub3JtYWwgY2VyZWJlbGx1bSAoQ0IpIHNhbXBsZSBpbmZvcm1hdGlvbgoKYGBge3J9CmNvdW50cyA8LSByZWFkLnRhYmxlKCIuL2RhdGEvR1NFMjAzMTc0L21STkFfcmF3X2NvdW50c19tYXRyaXgudHh0Iiwgc2VwPSJcdCIsIGhlYWRlcj1UUlVFKQptZXRhZGF0YSA8LSByZWFkLnRhYmxlKCIuL2RhdGEvR1NFMjAzMTc0L3NhbXBsZV9pbmZvcm1hdGlvbi50eHQiLCBzZXA9Ilx0IiwgaGVhZGVyPVRSVUUpCmBgYAoKIyBNb2RpZnkgZGF0YQoKUm93IGRhdGE6Ci0gQ29udmVydCBlbnNlbWJsIElEcyBpbnRvIGdlbmUgc3ltYm9scyBhbmQgZW50cmV6IElEcwoKQ291bnQgbWF0cml4OgotIFVzZSBjb2x1bW4gImlkIiBmb3Igcm93bmFtZXMgYW5kIHJlbW92ZSB0aGlzIGNvbHVtbgotIEtlZXAgb25seSBzYW1wbGVzIHdpdGggIm1fIiAoUk5BLVNlcSkKLSBNb2RpZnkgY29sdW1uIG5hbWVzCgpDb2x1bW4gZGF0YToKLSBjcmVhdGUgcm93bmFtZXMgaWRlbnRpY2FsIHRvIGNvdW50IG1hdHJpeCBzYW1wbGUgbmFtZXMKLSBrZWVwIG5lY2Nlc3NhcnkgY29sdW1ucwotIG1lcmdlIHN1Ymdyb3VwcyAiX3Byb2ciLCAiX3JlYyIgaW50byB0aGUgcmVzcGVjdGl2ZSBncm91cHMKCi0gcmVvcmRlciBzYW1wbGVzIG9mIHRoZSBjb3VudCBtYXRyaXggYmFzZWQgb24gY29sdW1uIGRhdGEKCgpgYGB7cn0KIyByb3cgZGF0YQplbnNlbWJsSURzIDwtIGdzdWIoIlxcLi4qIiwgIiIsIGNvdW50cyRpZCkKZ2VuZVN5bWJvbCA8LSBBbm5vdGF0aW9uRGJpOjptYXBJZHMob3JnLkhzLmVnLmRiLCBrZXlzID0gZW5zZW1ibElEcywga2V5dHlwZSA9ICJFTlNFTUJMIiwgY29sdW1uPSJTWU1CT0wiKQplbnRyZXpJRHMgPC0gQW5ub3RhdGlvbkRiaTo6bWFwSWRzKG9yZy5Icy5lZy5kYiwga2V5cyA9IGVuc2VtYmxJRHMsIGtleXR5cGUgPSAiRU5TRU1CTCIsIGNvbHVtbj0iRU5UUkVaSUQiKQoKcm93ZGF0YSA8LSBkYXRhLmZyYW1lKGVuc2VtYmxJRHMgPSBlbnNlbWJsSURzLCBnZW5lU3ltYm9sID0gZ2VuZVN5bWJvbCwgZW50cmV6SURzID0gZW50cmV6SURzLCByb3cubmFtZXMgPSBjb3VudHMkaWQpCiMgVmlldyhyb3dkYXRhKQoKIyBjb3VudCBkYXRhCnJvd25hbWVzKGNvdW50cykgPC0gY291bnRzWywxXQpjb3VudHMgPC0gY291bnRzWywtd2hpY2goY29sbmFtZXMoY291bnRzKT09ImlkIildCmNvdW50cyA8LSBjb3VudHNbLGdyZXBsKCJtXyIsIGNvbG5hbWVzKGNvdW50cykpXQpjb2xuYW1lcyhjb3VudHMpIDwtIGdzdWIoIl8uKiIsICIiLCBjb2xuYW1lcyhjb3VudHMpKQoKIyBWaWV3KGNvdW50cykKCiMgY29sdW1uIGRhdGEKY29sZGF0YV9pZCAgPC0gbWV0YWRhdGEkTGFiZWxfUk5BLnNlcS50eXBlLi5tUk5BLgpjb2xkYXRhX2lkWzE6OV0gPC0gcGFzdGUwKCIwIiwgY29sZGF0YV9pZFsxOjldKQpjb2xkYXRhX2lkIDwtIGdzdWIoIi0iLCAiIiwgY29sZGF0YV9pZCkKY29sZGF0YV9pZCA8LSBwYXN0ZTAoIlgiLCBjb2xkYXRhX2lkKQoKYWxsKGNvbGRhdGFfaWQgJWluJSBjb2xuYW1lcyhjb3VudHMpKQphbGwoY29sbmFtZXMoY291bnRzKSAlaW4lIGNvbGRhdGFfaWQpCgpjb2xkYXRhIDwtIG1ldGFkYXRhWyxjKCJMYWJlbF9STkEuc2VxLnR5cGUuLm1STkEuIiwiR3JvdXAiLCJCaW9zcGVjaW1lbi5pZCIsIlJlc2VhcmNoLnN1YmplY3QuaWQiLCJMb2NhdGlvbi5hbmQuYWRkaXRpb25hbC5pbmZvcm1hdGlvbiIpXQpjb2xkYXRhJEdyb3VwcyA8LSBjb2xkYXRhJEdyb3VwCmNvbGRhdGEkR3JvdXBzIDwtIGdzdWIoIl8uKiIsICIiLCBjb2xkYXRhJEdyb3VwcykKCnJvd25hbWVzKGNvbGRhdGEpIDwtIGNvbGRhdGFfaWQKCiMgVmlldyhjb2xkYXRhKQoKIyByZW9yZGVyIGNvdW50cyBiYXNlZCBvbiBjb2xkYXRhCmNvdW50cyA8LSBjb3VudHNbLGNvbGRhdGFfaWRdCmBgYAoKIyBDcmVhdGUgREVTZXEyIG9iamVjdAoKYGBge3J9CmRkcyA8LSBERVNlcURhdGFTZXRGcm9tTWF0cml4KGNvdW50RGF0YSA9IGNvdW50cywKICAgICAgICAgICAgICAgICAgICAgICAgICAgICAgY29sRGF0YSA9IGNvbGRhdGEsCiAgICAgICAgICAgICAgICAgICAgICAgICAgICAgIHJvd0RhdGEgPSByb3dkYXRhLAogICAgICAgICAgICAgICAgICAgICAgICAgICAgICBkZXNpZ249IH4gR3JvdXBzKQoKZGRzCiNzYXZlUkRTKGRkcywgIi4vZGF0YS9HU0UyMDMxNzQvR1NFMjAzMTc0X2Rkcy5SZHMiKQpgYGAKCiMjIERhdGEgb3ZlcnZpZXc6CgpDb3VudCBkYXRhIGNvbnNpc3RzIG9mIGByIG5jb2woY291bnRzKGRkcykpYCBjb2x1bW5zIChzYW1wbGVzKSBhbmQgYHIgbnJvdyhjb3VudHMoZGRzKSlgIHJvd3MgKHRyYW5zY3JpcHRzKS4KCk5yLiBvZiBzYW1wbGVzIHBlciBncm91cDoKCmBgYHtyfQp0YWJsZShhcy5mYWN0b3IoY29sRGF0YShkZHMpJEdyb3VwKSkKYGBgCgpOci4gb2Ygc2FtcGxlcyBwZXIgYmlnIGdyb3VwOgoKYGBge3J9CnRhYmxlKGNvbERhdGEoZGRzKSRHcm91cHMpCmBgYAoKIyBNaW5pbWFsIHByZS1maWx0ZXJpbmcKCkZpbHRlcmluZyBnZW5lcyB3aXRoIGxlc3MgdGhlbiAxMCBjb3VudHMgaW4gc3VtIGZvciBhbGwgc2FtcGxlcwoKYGBge3J9CmtlZXAgPC0gcm93U3Vtcyhjb3VudHMoZGRzKSkgPj0gMTAKZGRzIDwtIGRkc1trZWVwLF0KYGBgCgojIE5vcm1hbGl6YXRpb24gJiBsb2cxMCArMSAmIHN1YnNldCB0byBnZW5lcyBvZiBpbnRlcmVzdAoKLSBERVNlcTIgbm9ybWFsaXphdGlvbgotIGxvZzEwICsgMSB0cmFuc2Zvcm1hdGlvbgotIFN1YnNldHRpbmcgdG8gZ2VuZXMgb2YgaW50ZXJlc3QKCkdlbmVzIG9mIGludGVyZXN0IGFyZSBvYnRhaW5lZCBmcm9tIHRoZSBmb3VyIEtFR0cgcGF0aHdheXM6CgotIFNwaGluZ29saXBpZCBtZXRhYm9saXNtIChoc2EwMDYwMCkKLSBHbHljb3NwaGluZ29saXBpZCBiaW9zeW50aGVzaXMgLSBsYWN0byBhbmQgbmVvbGFjdG8gc2VyaWVzIChoc2EwMDYwMSkKLSBHbHljb3NwaGluZ29saXBpZCBiaW9zeW50aGVzaXMgLSBnbG9ibyBhbmQgaXNvZ2xvYm8gc2VyaWVzIChoc2EwMDYwMykKLSBHbHljb3NwaGluZ29saXBpZCBiaW9zeW50aGVzaXMgLSBnYW5nbGlvIHNlcmllcyAoaHNhMDA2MDQpCgpgYGB7cn0KZGRzIDwtIGVzdGltYXRlU2l6ZUZhY3RvcnMoZGRzKQoKdGFyZ2V0X2dlbmVzIDwtIHJlYWRSRFMoIi4vZGF0YS90YXJnZXRfZ2VuZXMuUmRzIikKbm9ybV9jb3VudHMgPC0gY291bnRzKGRkc1t3aGljaChyb3dEYXRhKGRkcykkZ2VuZVN5bWJvbCAlaW4lIGModGFyZ2V0X2dlbmVzJFNZTUJPTCwgIlNUOFNJQTMiKSksIF0sIG5vcm1hbGl6ZWQ9VFJVRSkKbm9ybV9jb3VudHMgPC0gbG9nMTAobm9ybV9jb3VudHMgKyAxKQoKcm93bmFtZXMobm9ybV9jb3VudHMpIDwtIHJvd0RhdGEoZGRzKSRnZW5lU3ltYm9sW3doaWNoKHJvd0RhdGEoZGRzKSRnZW5lU3ltYm9sICVpbiUgYyh0YXJnZXRfZ2VuZXMkU1lNQk9MLCAiU1Q4U0lBMyIpKV0KYGBgCgoKIyBPdmVydmlldyBnZW5lcyBvZiBpbnRlcmVzdAoKSGVhdG1hcCBzaG93aW5nIHRoZSBtZWRpYW4gbG9nMTAgbm9ybWFsaXplZCBnZW5lIGV4cHJlc3Npb24gcGVyIGdyb3VwLgoKYGBge3IsIGZpZy5oZWlnaHQ9IDE4fQpwaF9kZiA8LSB0KG5vcm1fY291bnRzKSAlPiUgYXMuZGF0YS5mcmFtZSgpCnBoX2RmJGdyb3VwIDwtIGNvbERhdGEoZGRzKSRHcm91cHMKCnBoX2RmICU8PiUKICAgIGdyb3VwX2J5KGdyb3VwKSAlPiUKICAgIGRwbHlyOjpzdW1tYXJpc2UoYWNyb3NzKGV2ZXJ5dGhpbmcoKSwgbWVkaWFuKSkgJT4lCiAgICBhcy5kYXRhLmZyYW1lKCkKCnJvd25hbWVzKHBoX2RmKSA8LSBwaF9kZiRncm91cApwaF9kZiA8LSBwaF9kZlssLXdoaWNoKGNvbG5hbWVzKHBoX2RmKT09Imdyb3VwIildCgpwaGVhdG1hcCh0KHBoX2RmKSwgYW5nbGVfY29sPSI0NSIsIGNlbGxoZWlnaHQ9MTIpCmBgYAoKIyBQQ0EKClJldHJpZXZpbmcgZ2VuZXMgZnJvbSBLRUdHIHBhdGh3YXlzCgpgYGB7cn0KZ2xfcHcxIDwtIGtlZ2dMaW5rKCJoc2EiLCJoc2EwMDYwMCIpICMgU3BoaW5nb2xpcGlkIG1ldGFib2xpc20KZ2xfcHcyIDwtIGtlZ2dMaW5rKCJoc2EiLCJoc2EwMDYwMSIpICMgR2x5Y29zcGhpbmdvbGlwaWQgYmlvc3ludGhlc2lzIC0gbGFjdG8gYW5kIG5lb2xhY3RvIHNlcmllcwpnbF9wdzMgPC0ga2VnZ0xpbmsoImhzYSIsImhzYTAwNjAzIikgIyBHbHljb3NwaGluZ29saXBpZCBiaW9zeW50aGVzaXMgLSBnbG9ibyBhbmQgaXNvZ2xvYm8gc2VyaWVzCmdsX3B3NCA8LSBrZWdnTGluaygiaHNhIiwiaHNhMDA2MDQiKSAjIEdseWNvc3BoaW5nb2xpcGlkIGJpb3N5bnRoZXNpcyAtIGdhbmdsaW8gc2VyaWVzCgppZHNfcHcxIDwtIGdzdWIoImhzYToiLCAiIiwgZ2xfcHcxKQpwdzFfc3ltYm9sSURzIDwtIEFubm90YXRpb25EYmk6Om1hcElkcyhvcmcuSHMuZWcuZGIsIGtleXMgPSBpZHNfcHcxLCBrZXl0eXBlID0gIkVOVFJFWklEIiwgY29sdW1uPSJTWU1CT0wiKQojVmlldyhwdzFfc3ltYm9sSURzKQoKaWRzX3B3MiA8LSBnc3ViKCJoc2E6IiwgIiIsIGdsX3B3MikKcHcyX3N5bWJvbElEcyA8LSBBbm5vdGF0aW9uRGJpOjptYXBJZHMob3JnLkhzLmVnLmRiLCBrZXlzID0gaWRzX3B3Miwga2V5dHlwZSA9ICJFTlRSRVpJRCIsIGNvbHVtbj0iU1lNQk9MIikKI1ZpZXcocHcyX3N5bWJvbElEcykKCmlkc19wdzMgPC0gZ3N1YigiaHNhOiIsICIiLCBnbF9wdzMpCnB3M19zeW1ib2xJRHMgPC0gQW5ub3RhdGlvbkRiaTo6bWFwSWRzKG9yZy5Icy5lZy5kYiwga2V5cyA9IGlkc19wdzMsIGtleXR5cGUgPSAiRU5UUkVaSUQiLCBjb2x1bW49IlNZTUJPTCIpCiNWaWV3KHB3M19zeW1ib2xJRHMpCgppZHNfcHc0IDwtIGdzdWIoImhzYToiLCAiIiwgZ2xfcHc0KQpwdzRfc3ltYm9sSURzIDwtIEFubm90YXRpb25EYmk6Om1hcElkcyhvcmcuSHMuZWcuZGIsIGtleXMgPSBpZHNfcHc0LCBrZXl0eXBlID0gIkVOVFJFWklEIiwgY29sdW1uPSJTWU1CT0wiKQpgYGAKCgojIyBQQ0EgYmFzZWQgb24gdGhlIG1vc3QgaW1wb3J0YW50IGtub3duIGVuenltZXMgaW4gZ2FuZ2xpb3NpZGUgYmlvc3ludGhlc2lzOiBTVDNHQUw1LCBTVDhTSUExLCBTVDhTSUE1LCBCM0dBTFQ0LCBCNEdBTE5UMSwgYW5kIEI0R0FMVDYKCmBgYHtyfQpwY2EgPC0gcHJjb21wKHQobm9ybV9jb3VudHNbYygiU1QzR0FMNSIsICJTVDhTSUExIiwgIlNUOFNJQTUiLCAiQjNHQUxUNCIsICJCNEdBTE5UMSIsICJCNEdBTFQ2IiksXSkpCgpnZ2JpcGxvdChwY2EsIGNob2ljZXMgPSAxOjIsIG9icy5zY2FsZSA9IDEsIHZhci5zY2FsZSA9IDEsCiAgICAgICAgIGdyb3VwcyA9IGNvbERhdGEoZGRzKSRHcm91cHMsIAogICAgICAgICBlbGxpcHNlID0gVCwgY2lyY2xlID0gRikgKwogIHNjYWxlX2NvbG9yX2Rpc2NyZXRlKG5hbWUgPSAnJykgKwogIHRoZW1lKGxlZ2VuZC5kaXJlY3Rpb24gPSAnaG9yaXpvbnRhbCcsIGxlZ2VuZC5wb3NpdGlvbiA9ICd0b3AnKQoKYGBgCgojIyBQQ0EgYmFzZWQgb24gZ2VuZXMgb2YgdGhlIFNwaGluZ29saXBpZCBtZXRhYm9saXNtIHBhdGh3YXkuCgpgYGB7cn0KcGNhIDwtIHByY29tcCh0KG5vcm1fY291bnRzW3doaWNoKHJvd25hbWVzKG5vcm1fY291bnRzKSAlaW4lIHB3MV9zeW1ib2xJRHMpLF0pKQoKZ2diaXBsb3QocGNhLCBjaG9pY2VzID0gMToyLCBvYnMuc2NhbGUgPSAxLCB2YXIuc2NhbGUgPSAxLAogICAgICAgICBncm91cHMgPSBjb2xEYXRhKGRkcykkR3JvdXBzLCAKICAgICAgICAgZWxsaXBzZSA9IFQsIGNpcmNsZSA9IEYsIHZhci5heGVzPUYpICsKICBzY2FsZV9jb2xvcl9kaXNjcmV0ZShuYW1lID0gJycpICsKICB0aGVtZShsZWdlbmQuZGlyZWN0aW9uID0gJ2hvcml6b250YWwnLCBsZWdlbmQucG9zaXRpb24gPSAndG9wJykKCmBgYAoKIyMgUENBIGJhc2VkIG9uIGdlbmVzIG9mIHRoZSBHbHljb3NwaGluZ29saXBpZCBiaW9zeW50aGVzaXMgLSBsYWN0byBhbmQgbmVvbGFjdG8gc2VyaWVzIHBhdGh3YXkuCgpgYGB7cn0KcGNhIDwtIHByY29tcCh0KG5vcm1fY291bnRzW3doaWNoKHJvd25hbWVzKG5vcm1fY291bnRzKSAlaW4lIHB3Ml9zeW1ib2xJRHMpLF0pKQoKZ2diaXBsb3QocGNhLCBjaG9pY2VzID0gMToyLCBvYnMuc2NhbGUgPSAxLCB2YXIuc2NhbGUgPSAxLAogICAgICAgICBncm91cHMgPSBjb2xEYXRhKGRkcykkR3JvdXBzLCAKICAgICAgICAgZWxsaXBzZSA9IFQsIGNpcmNsZSA9IEYsIHZhci5heGVzPUYpICsKICBzY2FsZV9jb2xvcl9kaXNjcmV0ZShuYW1lID0gJycpICsKICB0aGVtZShsZWdlbmQuZGlyZWN0aW9uID0gJ2hvcml6b250YWwnLCBsZWdlbmQucG9zaXRpb24gPSAndG9wJykKCmBgYAoKIyMgUENBIGJhc2VkIG9uIGdlbmVzIG9mIHRoZSBHbHljb3NwaGluZ29saXBpZCBiaW9zeW50aGVzaXMgLSBnbG9ibyBhbmQgaXNvZ2xvYm8gc2VyaWVzIHBhdGh3YXkuCgpgYGB7cn0KcGNhIDwtIHByY29tcCh0KG5vcm1fY291bnRzW3doaWNoKHJvd25hbWVzKG5vcm1fY291bnRzKSAlaW4lIHB3M19zeW1ib2xJRHMpLF0pKQoKZ2diaXBsb3QocGNhLCBjaG9pY2VzID0gMToyLCBvYnMuc2NhbGUgPSAxLCB2YXIuc2NhbGUgPSAxLAogICAgICAgICBncm91cHMgPSBjb2xEYXRhKGRkcykkR3JvdXBzLCAKICAgICAgICAgZWxsaXBzZSA9IFQsIGNpcmNsZSA9IEYsIHZhci5heGVzPUYpICsKICBzY2FsZV9jb2xvcl9kaXNjcmV0ZShuYW1lID0gJycpICsKICB0aGVtZShsZWdlbmQuZGlyZWN0aW9uID0gJ2hvcml6b250YWwnLCBsZWdlbmQucG9zaXRpb24gPSAndG9wJykKCmBgYAoKIyMgUENBIGJhc2VkIG9uIGdlbmVzIG9mIHRoZSBHbHljb3NwaGluZ29saXBpZCBiaW9zeW50aGVzaXMgLSBnYW5nbGlvIHNlcmllcyBwYXRod2F5LgoKYGBge3J9CnBjYSA8LSBwcmNvbXAodChub3JtX2NvdW50c1t3aGljaChyb3duYW1lcyhub3JtX2NvdW50cykgJWluJSBwdzRfc3ltYm9sSURzKSxdKSkKCmdnYmlwbG90KHBjYSwgY2hvaWNlcyA9IDE6Miwgb2JzLnNjYWxlID0gMSwgdmFyLnNjYWxlID0gMSwKICAgICAgICAgZ3JvdXBzID0gY29sRGF0YShkZHMpJEdyb3VwcywgCiAgICAgICAgIGVsbGlwc2UgPSBULCBjaXJjbGUgPSBGLCB2YXIuYXhlcz1GKSArCiAgc2NhbGVfY29sb3JfZGlzY3JldGUobmFtZSA9ICcnKSArCiAgdGhlbWUobGVnZW5kLmRpcmVjdGlvbiA9ICdob3Jpem9udGFsJywgbGVnZW5kLnBvc2l0aW9uID0gJ3RvcCcpCgpgYGAKCiMjIFBDQSBiYXNlZCBvbiBhbGwgZ2VuZXMgb2YgdGhlIGZvdXIgS0VHRyBwYXRod2F5cwoKYGBge3J9CiMgcGNhIDwtIHByY29tcCh0KG5vcm1fY291bnRzKSkKIyAKIyBkdHAgPC0gZGF0YS5mcmFtZSgnR3JvdXBzJyA9IGNvbERhdGEoZGRzKSRHcm91cHMsIHBjYSR4WywxOjNdKQojIHBsb3RseTo6cGxvdF9seShkdHAsIHggPSB+UEMxLCB5ID0gflBDMiwgeiA9IH5QQzMsIGNvbG9yID0gfkdyb3VwcykKCnBjYSA8LSBwcmNvbXAodChub3JtX2NvdW50cykpCgpnZ2JpcGxvdChwY2EsIGNob2ljZXMgPSAxOjIsIG9icy5zY2FsZSA9IDEsIHZhci5zY2FsZSA9IDEsCiAgICAgICAgIGdyb3VwcyA9IGNvbERhdGEoZGRzKSRHcm91cHMsIAogICAgICAgICBlbGxpcHNlID0gVCwgY2lyY2xlID0gRiwgdmFyLmF4ZXM9RikgKwogIHNjYWxlX2NvbG9yX2Rpc2NyZXRlKG5hbWUgPSAnJykgKwogIHRoZW1lKGxlZ2VuZC5kaXJlY3Rpb24gPSAnaG9yaXpvbnRhbCcsIGxlZ2VuZC5wb3NpdGlvbiA9ICd0b3AnKQpgYGAKCiMgRGlmZmVyZW50aWFsIEdlbmUgRXhwcmVzc2lvbiBBbmFseXNpcwoKYGBge3J9CkZEUiA8LSAwLjA1CgpkZHMgPC0gZGRzWyFkdXBsaWNhdGVkKHJvd0RhdGEoZGRzKSRlbnNlbWJsSURzKSxdCnJvd25hbWVzKGRkcykgPC0gcm93RGF0YShkZHMpJGVuc2VtYmxJRHMKIyBpZGVhbDo6aWRlYWwoZGRzKQoKY29sRGF0YShkZHMpJEdyb3VwcyA8LSByZWxldmVsKGNvbERhdGEoZGRzKSRHcm91cHMsICJTSEgiKQoKZGRzIDwtIERFU2VxMjo6REVTZXEoZGRzLCBwYXJhbGxlbCA9IFRSVUUpCgpyZXN1bHRzTmFtZXMoZGRzKQoKc3VtbWFyeShyZXN1bHRzKGRkcykpCmBgYAoKIyMgQ0IgdnMgU0hICgpgYGB7cn0KZGRzX3JlczEgPC0gcmVzdWx0cyhkZHMsIGFscGhhPUZEUiwgY29udHJhc3Q9YygiR3JvdXBzIiwgIkNCIiwgIlNISCIpKQoKc3VtbWFyeShkZHNfcmVzMSkKCmRkc19yZXMxX2RmIDwtIGFzLmRhdGEuZnJhbWUoZGRzX3JlczEpCgpkZHNfcmVzMV9kZiA8LSBkZHNfcmVzMV9kZltyb3duYW1lcyhkZHNfcmVzMV9kZikgJWluJSB0YXJnZXRfZ2VuZXMkRU5TRU1CTCxdCgpkZHNfcmVzMV9kZiA8LSByb3VuZChkZHNfcmVzMV9kZiwgNSkKCmRkc19yZXMxX2RmJEdlbmVTeW1ib2wgPC0gQW5ub3RhdGlvbkRiaTo6bWFwSWRzKG9yZy5Icy5lZy5kYiwga2V5cyA9IHJvd25hbWVzKGRkc19yZXMxX2RmKSwga2V5dHlwZSA9ICJFTlNFTUJMIiwgY29sdW1uPSJTWU1CT0wiKQoKRFQ6OmRhdGF0YWJsZShkZHNfcmVzMV9kZiwgY2FwdGlvbj0iR3JvdXBzX0NCX3ZzX1NISCwgREUgZ2VuZXMgKHJvdW5kZWQgdmFsdWVzKSIpCmBgYAoKIyMgR3JvdXAzIHZzIFNISAoKYGBge3J9CmRkc19yZXMyIDwtIHJlc3VsdHMoZGRzLCBhbHBoYT1GRFIsIGNvbnRyYXN0PWMoIkdyb3VwcyIsICJHcm91cDMiLCAiU0hIIikpCgpzdW1tYXJ5KGRkc19yZXMyKQoKZGRzX3JlczJfZGYgPC0gYXMuZGF0YS5mcmFtZShkZHNfcmVzMikKCmRkc19yZXMyX2RmIDwtIGRkc19yZXMyX2RmW3Jvd25hbWVzKGRkc19yZXMyX2RmKSAlaW4lIHRhcmdldF9nZW5lcyRFTlNFTUJMLF0KCmRkc19yZXMyX2RmIDwtIHJvdW5kKGRkc19yZXMyX2RmLCA1KQoKZGRzX3JlczJfZGYkR2VuZVN5bWJvbCA8LSBBbm5vdGF0aW9uRGJpOjptYXBJZHMob3JnLkhzLmVnLmRiLCBrZXlzID0gcm93bmFtZXMoZGRzX3JlczJfZGYpLCBrZXl0eXBlID0gIkVOU0VNQkwiLCBjb2x1bW49IlNZTUJPTCIpCgpEVDo6ZGF0YXRhYmxlKGRkc19yZXMyX2RmLCBjYXB0aW9uPSJHcm91cHNfR3JvdXAzX3ZzX1NISCwgREUgZ2VuZXMgKHJvdW5kZWQgdmFsdWVzKSIpCmBgYAoKIyMgR3JvdXA0IHZzIFNISAoKYGBge3J9CmRkc19yZXMzIDwtIHJlc3VsdHMoZGRzLCBhbHBoYT1GRFIsIGNvbnRyYXN0PWMoIkdyb3VwcyIsICJHcm91cDQiLCAiU0hIIikpCgpzdW1tYXJ5KGRkc19yZXMzKQoKZGRzX3JlczNfZGYgPC0gYXMuZGF0YS5mcmFtZShkZHNfcmVzMykKCmRkc19yZXMzX2RmIDwtIGRkc19yZXMzX2RmW3Jvd25hbWVzKGRkc19yZXMzX2RmKSAlaW4lIHRhcmdldF9nZW5lcyRFTlNFTUJMLF0KCmRkc19yZXMzX2RmIDwtIHJvdW5kKGRkc19yZXMzX2RmLCA1KQoKZGRzX3JlczNfZGYkR2VuZVN5bWJvbCA8LSBBbm5vdGF0aW9uRGJpOjptYXBJZHMob3JnLkhzLmVnLmRiLCBrZXlzID0gcm93bmFtZXMoZGRzX3JlczNfZGYpLCBrZXl0eXBlID0gIkVOU0VNQkwiLCBjb2x1bW49IlNZTUJPTCIpCgpEVDo6ZGF0YXRhYmxlKGRkc19yZXMzX2RmLCBjYXB0aW9uPSJHcm91cHNfR3JvdXA0X3ZzX1NISCwgREUgZ2VuZXMgKHJvdW5kZWQgdmFsdWVzKSIpCmBgYAoKIyMgV05UIHZzIFNISAoKYGBge3J9CmRkc19yZXM0IDwtIHJlc3VsdHMoZGRzLCBhbHBoYT1GRFIsIGNvbnRyYXN0PWMoIkdyb3VwcyIsICJXTlQiLCAiU0hIIikpCgpzdW1tYXJ5KGRkc19yZXM0KQoKZGRzX3JlczRfZGYgPC0gYXMuZGF0YS5mcmFtZShkZHNfcmVzNCkKCmRkc19yZXM0X2RmIDwtIGRkc19yZXM0X2RmW3Jvd25hbWVzKGRkc19yZXM0X2RmKSAlaW4lIHRhcmdldF9nZW5lcyRFTlNFTUJMLF0KCmRkc19yZXM0X2RmIDwtIHJvdW5kKGRkc19yZXM0X2RmLCA1KQoKZGRzX3JlczRfZGYkR2VuZVN5bWJvbCA8LSBBbm5vdGF0aW9uRGJpOjptYXBJZHMob3JnLkhzLmVnLmRiLCBrZXlzID0gcm93bmFtZXMoZGRzX3JlczRfZGYpLCBrZXl0eXBlID0gIkVOU0VNQkwiLCBjb2x1bW49IlNZTUJPTCIpCgpEVDo6ZGF0YXRhYmxlKGRkc19yZXM0X2RmLCBjYXB0aW9uPSJHcm91cHNfV05UX3ZzX1NISCwgREUgZ2VuZXMgKHJvdW5kZWQgdmFsdWVzKSIpCmBgYAoKR2VuZXJhdGluZyBsaXN0IHdpdGggYWxsIGdlbmVzIHdoZXJlIFAgYWRqdXN0ZWQgdmFsdWUgPCAwLjA1IGluIDMgb3IgbW9yZSBjb21wYXJpc29ucyAKCmBgYHtyfQpnZW5lbGlzdCA8LWMoCiAgZGRzX3JlczFfZGZbZGRzX3JlczFfZGYkcGFkajw9MC4wNSwiR2VuZVN5bWJvbCJdLAogIGRkc19yZXMyX2RmW2Rkc19yZXMyX2RmJHBhZGo8PTAuMDUsIkdlbmVTeW1ib2wiXSwKICBkZHNfcmVzM19kZltkZHNfcmVzM19kZiRwYWRqPD0wLjA1LCJHZW5lU3ltYm9sIl0sCiAgZGRzX3JlczRfZGZbZGRzX3JlczRfZGYkcGFkajw9MC4wNSwiR2VuZVN5bWJvbCJdCikKCmdlbmVsaXN0X2ZyZXEgPC0gdGFibGUoZ2VuZWxpc3RbIWlzLm5hKGdlbmVsaXN0KV0pCmdlbmVsaXN0X2ZyZXEgPC0gZ2VuZWxpc3RfZnJlcVtnZW5lbGlzdF9mcmVxPjJdCmdlbmVsaXN0IDwtIG5hbWVzKGdlbmVsaXN0X2ZyZXEpCmdlbmVsaXN0CmBgYAoKYGBge3J9CmYgPC0gcmVwKHNlcV9sZW4oY2VpbGluZyhsZW5ndGgoZ2VuZWxpc3QpIC8gMikpLGVhY2ggPSAyLGxlbmd0aC5vdXQgPSBsZW5ndGgoZ2VuZWxpc3QpKQoKZm9yKGkgaW4gdW5pcXVlKGYpKXsKICBwIDwtIGNhcmV0OjpmZWF0dXJlUGxvdCh4ID0gdChub3JtX2NvdW50cylbLGdlbmVsaXN0W3doaWNoKGYgJWluJSBpKV1dLAogICAgICAgICAgICB5ID0gY29sRGF0YShkZHMpJEdyb3VwcywKICAgICAgICAgICAgcGxvdCA9ICJib3giLAogICAgICAgICAgICBzY2FsZXMgPSBsaXN0KHggPSBsaXN0KHJlbGF0aW9uPSJmcmVlIiwgcm90PTkwKSwKICAgICAgICAgICAgICAgICAgICAgICAgICB5ID0gbGlzdChyZWxhdGlvbj0iZnJlZSIpKSkKICBwcmludChwKQp9CgoKYGBgCgojIFR3byBnZW5lIHNpZ25hdHVyZSBTVDhTSUExICYgQjRHQUxOVAoKYGBge3J9CnNpZ19kZiA8LSB0KG5vcm1fY291bnRzW2MoIlNUOFNJQTEiLCAiQjRHQUxOVDEiKSxdKSAlPiUgYXMuZGF0YS5mcmFtZSgpCnNpZ19kZiRTVDhTSUExX0I0R0FMTlQxIDwtIHNpZ19kZiRTVDhTSUExICsgc2lnX2RmJEI0R0FMTlQxCnNpZ19kZiRHcm91cHMgPC0gYXMuZmFjdG9yKGNvbERhdGEoZGRzKSRHcm91cHMpCnNpZ19kZiRTYW1wbGUgPC0gcm93bmFtZXMoc2lnX2RmKQoKIyBzaWdfZGZfbG9uZyA8LSBzaWdfZGYgJT4lCiMgICAgICAgZ3JvdXBfYnkoR3JvdXBzKSAlPiUKIyAgICAgICB0aWR5cjo6cGl2b3RfbG9uZ2VyKGNvbG5hbWVzKGRwbHlyOjpzZWxlY3Qoc2lnX2RmLCAhYygiU2FtcGxlIiwgIkdyb3VwcyIpKSksIG5hbWVzX3RvID0gImdlbmUiLCB2YWx1ZXNfdG8gPSAidmFsdWUiKQoKcCA8LSBnZ3Bsb3Qoc2lnX2RmLCBhZXMoeCA9IEdyb3VwcywgeSA9IFNUOFNJQTFfQjRHQUxOVDEsIGZpbGwgPSBHcm91cHMpKSArCiAgZ2VvbV9ib3hwbG90KCkgKwogIHNjYWxlX2ZpbGxfdmlyaWRpcyhkaXNjcmV0ZSA9IFRSVUUsIGFscGhhID0gMC42KSArCiAgZ2VvbV9qaXR0ZXIoY29sb3IgPSAiYmxhY2siLAogICAgICAgICAgICAgIHNpemUgPSAwLjQsCiAgICAgICAgICAgICAgYWxwaGEgPSAwLjkpICsKICB0aGVtZV9jbGFzc2ljKCkgKwogIHRoZW1lKGxlZ2VuZC5wb3NpdGlvbiA9ICJub25lIiwKICAgICAgICBwbG90LnRpdGxlID0gZWxlbWVudF90ZXh0KHNpemUgPSAxMSkpICsKICBnZ3RpdGxlKCJUd28gZ2VuZSBzaWduYXR1cmUiKSArCiAgeWxhYigibG9nMTAoIFNUOFNJQTEgKyBCNEdBTE5UMSApIikgKwogIHhsYWIoIiIpCnAKYGBgCgojIyBUd28gZ2VuZSBzaWduYXR1cmUgd2l0aCBOZXVyb2JsYXN0b21hCgpgYGB7cn0KbmJTRSA8LSByZWFkUkRTKCIuL2RhdGEveGVuYWJyb3dzZXIvTkJfZ2VuZV9leHBlY3RlZF9jb3VudC5SZHMiKQoKcm93Lm5hbWVzKG5iU0UpIDwtIGdzdWIoIlxcLi4qIiwgIiIsIHJvd25hbWVzKG5iU0UpKQphc3NheXMobmJTRSkkaW50ZWdlckNvdW50cyA8LSByb3VuZCgoKDJeYXNzYXlzKG5iU0UpJGNvdW50cykgLSAxKSwgMCkKCmNvbW1vbl9nZW5lcyA8LSBpbnRlcnNlY3Qocm93bmFtZXMoZGRzKSwgcm93bmFtZXMobmJTRSkpCmNvdW50c19kZHMgPC0gY291bnRzKGRkc1tjb21tb25fZ2VuZXMsXSwgbm9ybWFsaXplZD1GQUxTRSkKY291bnRzX25iIDwtIGFzc2F5cyhuYlNFKSRpbnRlZ2VyQ291bnRzW2NvbW1vbl9nZW5lcyxdCmNvdW50c19kZHNfbmIgPC0gY2JpbmQoY291bnRzX2RkcywgY291bnRzX25iKQoKCmNvbGRhdGFfZGRzX25iIDwtIGRhdGEuZnJhbWUoR3JvdXA9Yyhjb2xEYXRhKGRkcykkR3JvdXBzLCBhcy5mYWN0b3IoY29sRGF0YShuYlNFKSRkZXRhaWxlZF9jYXRlZ29yeSkpKQoKbGV2ZWxzKGNvbGRhdGFfZGRzX25iJEdyb3VwKSA8LSBjKCJTSEgiLCJDQiIsIkdyb3VwMyIsIkdyb3VwNCIsIldOVCIsIk5CTCIpCgpkZHNfbmJsIDwtIERFU2VxMjo6REVTZXFEYXRhU2V0RnJvbU1hdHJpeChjb3VudHNfZGRzX25iLCBjb2xkYXRhX2Rkc19uYiwgZGVzaWduPX5Hcm91cCkKCmRkc19uYmwgPC0gZXN0aW1hdGVTaXplRmFjdG9ycyhkZHNfbmJsKQpub3JtX2NvdW50cyA8LSBjb3VudHMoZGRzX25ibFt3aGljaChyb3duYW1lcyhkZHNfbmJsKSAlaW4lIGModGFyZ2V0X2dlbmVzJEVOU0VNQkwpKSwgXSwgbm9ybWFsaXplZD1UUlVFKQpub3JtX2NvdW50cyA8LSBsb2cxMChub3JtX2NvdW50cyArIDEpCgpyb3duYW1lcyhub3JtX2NvdW50cykgPC0gQW5ub3RhdGlvbkRiaTo6bWFwSWRzKG9yZy5Icy5lZy5kYiwga2V5cyA9IHJvd25hbWVzKG5vcm1fY291bnRzKSwga2V5dHlwZSA9ICJFTlNFTUJMIiwgY29sdW1uPSJTWU1CT0wiKQpgYGAKCgpgYGB7cn0Kc2lnX2RmIDwtIHQobm9ybV9jb3VudHNbYygiU1Q4U0lBMSIsICJCNEdBTE5UMSIpLF0pICU+JSBhcy5kYXRhLmZyYW1lKCkKc2lnX2RmJFNUOFNJQTFfQjRHQUxOVDEgPC0gc2lnX2RmJFNUOFNJQTEgKyBzaWdfZGYkQjRHQUxOVDEKc2lnX2RmJEdyb3VwIDwtIGFzLmZhY3Rvcihjb2xEYXRhKGRkc19uYmwpJEdyb3VwKQpzaWdfZGYkU2FtcGxlIDwtIHJvd25hbWVzKHNpZ19kZikKCiMgc2lnX2RmX2xvbmcgPC0gc2lnX2RmICU+JQojICAgICAgIGdyb3VwX2J5KEdyb3VwcykgJT4lCiMgICAgICAgdGlkeXI6OnBpdm90X2xvbmdlcihjb2xuYW1lcyhkcGx5cjo6c2VsZWN0KHNpZ19kZiwgIWMoIlNhbXBsZSIsICJHcm91cHMiKSkpLCBuYW1lc190byA9ICJnZW5lIiwgdmFsdWVzX3RvID0gInZhbHVlIikKCnAgPC0gZ2dwbG90KHNpZ19kZiwgYWVzKHggPSBHcm91cCwgeSA9IFNUOFNJQTFfQjRHQUxOVDEsIGZpbGwgPSBHcm91cCkpICsKICBnZW9tX2JveHBsb3QoKSArCiAgc2NhbGVfZmlsbF92aXJpZGlzKGRpc2NyZXRlID0gVFJVRSwgYWxwaGEgPSAwLjYpICsKICBnZW9tX2ppdHRlcihjb2xvciA9ICJibGFjayIsCiAgICAgICAgICAgICAgc2l6ZSA9IDAuNCwKICAgICAgICAgICAgICBhbHBoYSA9IDAuOSkgKwogIHRoZW1lX2NsYXNzaWMoKSArCiAgdGhlbWUobGVnZW5kLnBvc2l0aW9uID0gIm5vbmUiLAogICAgICAgIHBsb3QudGl0bGUgPSBlbGVtZW50X3RleHQoc2l6ZSA9IDExKSkgKwogIGdndGl0bGUoIlR3byBnZW5lIHNpZ25hdHVyZSIpICsKICB5bGFiKCJsb2cxMCggU1Q4U0lBMSArIEI0R0FMTlQxICkiKSArCiAgeGxhYigiIikKcApgYGAKCmBgYHtyfQpybShuYlNFKQpybShjb3VudHNfZGRzKQpybShjb3VudHNfbmIpCnJtKGRkc19uYmwpCnJtKGNvdW50c19kZHNfbmIpCmdjKCkKYGBgCgoKIyBUd28gZ2VuZSBzaWduYXR1cmUgb2YgTUIgY29tYmluZWQgd2l0aCBvdGhlciBkYXRhIHNldHMKCiMjIEdURXggKyBNQgoKYGBge3J9Cmd0ZXhTRSA8LSByZWFkUkRTKCIuL2RhdGEveGVuYWJyb3dzZXIvR1RFeF9nZW5lX2V4cGVjdGVkX2NvdW50LlJkcyIpCgpyb3cubmFtZXMoZ3RleFNFKSA8LSBnc3ViKCJcXC4uKiIsICIiLCByb3duYW1lcyhndGV4U0UpKQphc3NheXMoZ3RleFNFKSRpbnRlZ2VyQ291bnRzIDwtIHJvdW5kKCgoMl5hc3NheXMoZ3RleFNFKSRjb3VudHMpIC0gMSksIDApCgpjb21tb25fZ2VuZXMgPC0gaW50ZXJzZWN0KHJvd25hbWVzKGRkcyksIHJvd25hbWVzKGd0ZXhTRSkpCmNvdW50c19kZHMgPC0gY291bnRzKGRkc1tjb21tb25fZ2VuZXMsXSwgbm9ybWFsaXplZD1GQUxTRSkKY291bnRzX2d0ZXggPC0gYXNzYXlzKGd0ZXhTRSkkaW50ZWdlckNvdW50c1tjb21tb25fZ2VuZXMsXQpjb3VudHNfZGRzX2d0ZXggPC0gY2JpbmQoY291bnRzX2RkcywgY291bnRzX2d0ZXgpCgpjb2xEYXRhKGd0ZXhTRSkkY2F0ZWdvcnlfZXh0ZW5kZWQgPC0gcGFzdGUwKGNvbERhdGEoZ3RleFNFKSRkZXRhaWxlZF9jYXRlZ29yeSwgIiAoIiwgY29sRGF0YShndGV4U0UpJFhfc2FtcGxlX3R5cGUsICIpIikKY29sZGF0YV9kZHNfZ3RleCA8LSBkYXRhLmZyYW1lKEdyb3VwPWMoY29sRGF0YShkZHMpJEdyb3VwcywgYXMuZmFjdG9yKGNvbERhdGEoZ3RleFNFKSRjYXRlZ29yeV9leHRlbmRlZCkpKQoKI2xldmVscyhjb2xkYXRhX2Rkc19uYiRHcm91cCkgPC0gYygiU0hIIiwiQ0IiLCJHcm91cDMiLCJHcm91cDQiLCJXTlQiLCJOQkwiKQoKZGRzX2d0ZXggPC0gREVTZXEyOjpERVNlcURhdGFTZXRGcm9tTWF0cml4KGNvdW50c19kZHNfZ3RleCwgY29sZGF0YV9kZHNfZ3RleCwgZGVzaWduPX5Hcm91cCkKCmRkc19ndGV4IDwtIGVzdGltYXRlU2l6ZUZhY3RvcnMoZGRzX2d0ZXgpCm5vcm1fY291bnRzIDwtIGNvdW50cyhkZHNfZ3RleFt3aGljaChyb3duYW1lcyhkZHNfZ3RleCkgJWluJSBjKHRhcmdldF9nZW5lcyRFTlNFTUJMKSksIF0sIG5vcm1hbGl6ZWQ9VFJVRSkKbm9ybV9jb3VudHMgPC0gbG9nMTAobm9ybV9jb3VudHMgKyAxKQoKcm93bmFtZXMobm9ybV9jb3VudHMpIDwtIEFubm90YXRpb25EYmk6Om1hcElkcyhvcmcuSHMuZWcuZGIsIGtleXMgPSByb3duYW1lcyhub3JtX2NvdW50cyksIGtleXR5cGUgPSAiRU5TRU1CTCIsIGNvbHVtbj0iU1lNQk9MIikKYGBgCgpgYGB7ciwgZmlnLmhlaWdodD0gMTh9Cm5vcm1fY291bnRzX3N1YiA8LSB0KG5vcm1fY291bnRzW2MoIlNUM0dBTDUiLCAiU1Q4U0lBMSIsICJTVDhTSUE1IiwgIkIzR0FMVDQiLCAiQjRHQUxOVDEiLCAiQjRHQUxUNiIpLF0pICU+JSBhcy5kYXRhLmZyYW1lKCkKbm9ybV9jb3VudHNfc3ViJFNUOFNJQTFfQjRHQUxOVDEgPC0gbm9ybV9jb3VudHNfc3ViJFNUOFNJQTEgKyBub3JtX2NvdW50c19zdWIkQjRHQUxOVDEKbm9ybV9jb3VudHNfc3ViJEdyb3VwIDwtIGNvbERhdGEoZGRzX2d0ZXgpJEdyb3VwCgpub3JtX2NvdW50c19zdWIgJTw+JQogICAgZ3JvdXBfYnkoR3JvdXApICU+JQogICAgZHBseXI6OnN1bW1hcmlzZShhY3Jvc3MoZXZlcnl0aGluZygpLCBtZWRpYW4pKSAlPiUKICAgIGFzLmRhdGEuZnJhbWUoKQoKcm93bmFtZXMobm9ybV9jb3VudHNfc3ViKSA8LSBub3JtX2NvdW50c19zdWIkR3JvdXAKbm9ybV9jb3VudHNfc3ViIDwtIG5vcm1fY291bnRzX3N1YlssLXdoaWNoKGNvbG5hbWVzKG5vcm1fY291bnRzX3N1Yik9PSJHcm91cCIpXQoKbm9ybV9jb3VudHNfc3ViIDwtIG5vcm1fY291bnRzX3N1YltvcmRlcihub3JtX2NvdW50c19zdWIkU1Q4U0lBMV9CNEdBTE5UMSwgZGVjcmVhc2luZyA9IFRSVUUpLF0KCnBoZWF0bWFwKG5vcm1fY291bnRzX3N1YiwgYW5nbGVfY29sPSI0NSIsIGNlbGxoZWlnaHQ9MTIsIGNsdXN0ZXJfcm93cyA9IEZBTFNFLCBjbHVzdGVyX2NvbHMgPSBGQUxTRSkKYGBgCgpgYGB7cn0Kcm0oZ3RleFNFKQpybShjb3VudHNfZGRzKQpybShjb3VudHNfZ3RleCkKcm0oY291bnRzX2Rkc19ndGV4KQpybShub3JtX2NvdW50cykKcm0obm9ybV9jb3VudHNfc3ViKQpybShkZHNfZ3RleCkKZ2MoKQpgYGAKCiMjIFRBUkdFVCArIE1CCgpgYGB7cn0KdGFyZ2V0U0UgPC0gcmVhZFJEUygiLi9kYXRhL3hlbmFicm93c2VyL1RBUkdFVF9nZW5lX2V4cGVjdGVkX2NvdW50LlJkcyIpCgpyb3cubmFtZXModGFyZ2V0U0UpIDwtIGdzdWIoIlxcLi4qIiwgIiIsIHJvd25hbWVzKHRhcmdldFNFKSkKYXNzYXlzKHRhcmdldFNFKSRpbnRlZ2VyQ291bnRzIDwtIHJvdW5kKCgoMl5hc3NheXModGFyZ2V0U0UpJGNvdW50cykgLSAxKSwgMCkKCmNvbW1vbl9nZW5lcyA8LSBpbnRlcnNlY3Qocm93bmFtZXMoZGRzKSwgcm93bmFtZXModGFyZ2V0U0UpKQpjb3VudHNfZGRzIDwtIGNvdW50cyhkZHNbY29tbW9uX2dlbmVzLF0sIG5vcm1hbGl6ZWQ9RkFMU0UpCmNvdW50c190YXJnZXQgPC0gYXNzYXlzKHRhcmdldFNFKSRpbnRlZ2VyQ291bnRzW2NvbW1vbl9nZW5lcyxdCmNvdW50c19kZHNfdGFyZ2V0IDwtIGNiaW5kKGNvdW50c19kZHMsIGNvdW50c190YXJnZXQpCgpjb2xEYXRhKHRhcmdldFNFKSRjYXRlZ29yeV9leHRlbmRlZCA8LSBwYXN0ZTAoY29sRGF0YSh0YXJnZXRTRSkkZGV0YWlsZWRfY2F0ZWdvcnksICIgKCIsIGNvbERhdGEodGFyZ2V0U0UpJFhfc2FtcGxlX3R5cGUsICIpIikKY29sZGF0YV9kZHNfdGFyZ2V0IDwtIGRhdGEuZnJhbWUoR3JvdXA9Yyhjb2xEYXRhKGRkcykkR3JvdXBzLCBhcy5mYWN0b3IoY29sRGF0YSh0YXJnZXRTRSkkY2F0ZWdvcnlfZXh0ZW5kZWQpKSkKCiNsZXZlbHMoY29sZGF0YV9kZHNfbmIkR3JvdXApIDwtIGMoIlNISCIsIkNCIiwiR3JvdXAzIiwiR3JvdXA0IiwiV05UIiwiTkJMIikKCmRkc190YXJnZXQgPC0gREVTZXEyOjpERVNlcURhdGFTZXRGcm9tTWF0cml4KGNvdW50c19kZHNfdGFyZ2V0LCBjb2xkYXRhX2Rkc190YXJnZXQsIGRlc2lnbj1+R3JvdXApCgpkZHNfdGFyZ2V0IDwtIGVzdGltYXRlU2l6ZUZhY3RvcnMoZGRzX3RhcmdldCkKbm9ybV9jb3VudHMgPC0gY291bnRzKGRkc190YXJnZXRbd2hpY2gocm93bmFtZXMoZGRzX3RhcmdldCkgJWluJSBjKHRhcmdldF9nZW5lcyRFTlNFTUJMKSksIF0sIG5vcm1hbGl6ZWQ9VFJVRSkKbm9ybV9jb3VudHMgPC0gbG9nMTAobm9ybV9jb3VudHMgKyAxKQoKcm93bmFtZXMobm9ybV9jb3VudHMpIDwtIEFubm90YXRpb25EYmk6Om1hcElkcyhvcmcuSHMuZWcuZGIsIGtleXMgPSByb3duYW1lcyhub3JtX2NvdW50cyksIGtleXR5cGUgPSAiRU5TRU1CTCIsIGNvbHVtbj0iU1lNQk9MIikKYGBgCgpgYGB7ciwgZmlnLmhlaWdodD0gMTgsICwgZmlnLndpZHRoID0gMTh9Cm5vcm1fY291bnRzX3N1YiA8LSB0KG5vcm1fY291bnRzW2MoIlNUM0dBTDUiLCAiU1Q4U0lBMSIsICJTVDhTSUE1IiwgIkIzR0FMVDQiLCAiQjRHQUxOVDEiLCAiQjRHQUxUNiIpLF0pICU+JSBhcy5kYXRhLmZyYW1lKCkKbm9ybV9jb3VudHNfc3ViJFNUOFNJQTFfQjRHQUxOVDEgPC0gbm9ybV9jb3VudHNfc3ViJFNUOFNJQTEgKyBub3JtX2NvdW50c19zdWIkQjRHQUxOVDEKbm9ybV9jb3VudHNfc3ViJEdyb3VwIDwtIGNvbERhdGEoZGRzX3RhcmdldCkkR3JvdXAKCm5vcm1fY291bnRzX3N1YiAlPD4lCiAgICBncm91cF9ieShHcm91cCkgJT4lCiAgICBkcGx5cjo6c3VtbWFyaXNlKGFjcm9zcyhldmVyeXRoaW5nKCksIG1lZGlhbikpICU+JQogICAgYXMuZGF0YS5mcmFtZSgpCgpyb3duYW1lcyhub3JtX2NvdW50c19zdWIpIDwtIG5vcm1fY291bnRzX3N1YiRHcm91cApub3JtX2NvdW50c19zdWIgPC0gbm9ybV9jb3VudHNfc3ViWywtd2hpY2goY29sbmFtZXMobm9ybV9jb3VudHNfc3ViKT09Ikdyb3VwIildCgpub3JtX2NvdW50c19zdWIgPC0gbm9ybV9jb3VudHNfc3ViW29yZGVyKG5vcm1fY291bnRzX3N1YiRTVDhTSUExX0I0R0FMTlQxLCBkZWNyZWFzaW5nID0gVFJVRSksXQoKcGhlYXRtYXAobm9ybV9jb3VudHNfc3ViLCBhbmdsZV9jb2w9IjQ1IiwgY2VsbGhlaWdodD0xMiwgY2x1c3Rlcl9yb3dzID0gRkFMU0UsIGNsdXN0ZXJfY29scyA9IEZBTFNFKQpgYGAKCmBgYHtyfQpybSh0YXJnZXRTRSkKcm0oY291bnRzX2RkcykKcm0oY291bnRzX3RhcmdldCkKcm0oY291bnRzX2Rkc190YXJnZXQpCnJtKG5vcm1fY291bnRzKQpybShub3JtX2NvdW50c19zdWIpCnJtKGRkc190YXJnZXQpCmdjKCkKYGBgCgojIyBUQ0dBICsgTUIKCmBgYHtyfQp0Y2dhU0UgPC0gcmVhZFJEUygiLi9kYXRhL3hlbmFicm93c2VyL1RDR0FfZ2VuZV9leHBlY3RlZF9jb3VudC5SZHMiKQoKcm93Lm5hbWVzKHRjZ2FTRSkgPC0gZ3N1YigiXFwuLioiLCAiIiwgcm93bmFtZXModGNnYVNFKSkKYXNzYXlzKHRjZ2FTRSkkaW50ZWdlckNvdW50cyA8LSByb3VuZCgoKDJeYXNzYXlzKHRjZ2FTRSkkY291bnRzKSAtIDEpLCAwKQoKY29tbW9uX2dlbmVzIDwtIGludGVyc2VjdChyb3duYW1lcyhkZHMpLCByb3duYW1lcyh0Y2dhU0UpKQpjb3VudHNfZGRzIDwtIGNvdW50cyhkZHNbY29tbW9uX2dlbmVzLF0sIG5vcm1hbGl6ZWQ9RkFMU0UpCmNvdW50c190Y2dhIDwtIGFzc2F5cyh0Y2dhU0UpJGludGVnZXJDb3VudHNbY29tbW9uX2dlbmVzLF0KY291bnRzX2Rkc190Y2dhIDwtIGNiaW5kKGNvdW50c19kZHMsIGNvdW50c190Y2dhKQoKY29sRGF0YSh0Y2dhU0UpJGNhdGVnb3J5X2V4dGVuZGVkIDwtIHBhc3RlMChjb2xEYXRhKHRjZ2FTRSkkZGV0YWlsZWRfY2F0ZWdvcnksICIgKCIsIGNvbERhdGEodGNnYVNFKSRYX3NhbXBsZV90eXBlLCAiKSIpCmNvbGRhdGFfZGRzX3RjZ2EgPC0gZGF0YS5mcmFtZShHcm91cD1jKGNvbERhdGEoZGRzKSRHcm91cHMsIGFzLmZhY3Rvcihjb2xEYXRhKHRjZ2FTRSkkY2F0ZWdvcnlfZXh0ZW5kZWQpKSkKCiNsZXZlbHMoY29sZGF0YV9kZHNfbmIkR3JvdXApIDwtIGMoIlNISCIsIkNCIiwiR3JvdXAzIiwiR3JvdXA0IiwiV05UIiwiTkJMIikKCmRkc190Y2dhIDwtIERFU2VxMjo6REVTZXFEYXRhU2V0RnJvbU1hdHJpeChjb3VudHNfZGRzX3RjZ2EsIGNvbGRhdGFfZGRzX3RjZ2EsIGRlc2lnbj1+R3JvdXApCgpkZHNfdGNnYSA8LSBlc3RpbWF0ZVNpemVGYWN0b3JzKGRkc190Y2dhKQpub3JtX2NvdW50cyA8LSBjb3VudHMoZGRzX3RjZ2Fbd2hpY2gocm93bmFtZXMoZGRzX3RjZ2EpICVpbiUgYyh0YXJnZXRfZ2VuZXMkRU5TRU1CTCkpLCBdLCBub3JtYWxpemVkPVRSVUUpCm5vcm1fY291bnRzIDwtIGxvZzEwKG5vcm1fY291bnRzICsgMSkKCnJvd25hbWVzKG5vcm1fY291bnRzKSA8LSBBbm5vdGF0aW9uRGJpOjptYXBJZHMob3JnLkhzLmVnLmRiLCBrZXlzID0gcm93bmFtZXMobm9ybV9jb3VudHMpLCBrZXl0eXBlID0gIkVOU0VNQkwiLCBjb2x1bW49IlNZTUJPTCIpCmBgYAoKYGBge3IsIGZpZy5oZWlnaHQ9IDE4fQpub3JtX2NvdW50c19zdWIgPC0gdChub3JtX2NvdW50c1tjKCJTVDNHQUw1IiwgIlNUOFNJQTEiLCAiU1Q4U0lBNSIsICJCM0dBTFQ0IiwgIkI0R0FMTlQxIiwgIkI0R0FMVDYiKSxdKSAlPiUgYXMuZGF0YS5mcmFtZSgpCm5vcm1fY291bnRzX3N1YiRTVDhTSUExX0I0R0FMTlQxIDwtIG5vcm1fY291bnRzX3N1YiRTVDhTSUExICsgbm9ybV9jb3VudHNfc3ViJEI0R0FMTlQxCm5vcm1fY291bnRzX3N1YiRHcm91cCA8LSBjb2xEYXRhKGRkc190Y2dhKSRHcm91cAoKbm9ybV9jb3VudHNfc3ViICU8PiUKICAgIGdyb3VwX2J5KEdyb3VwKSAlPiUKICAgIGRwbHlyOjpzdW1tYXJpc2UoYWNyb3NzKGV2ZXJ5dGhpbmcoKSwgbWVkaWFuKSkgJT4lCiAgICBhcy5kYXRhLmZyYW1lKCkKCnJvd25hbWVzKG5vcm1fY291bnRzX3N1YikgPC0gbm9ybV9jb3VudHNfc3ViJEdyb3VwCm5vcm1fY291bnRzX3N1YiA8LSBub3JtX2NvdW50c19zdWJbLC13aGljaChjb2xuYW1lcyhub3JtX2NvdW50c19zdWIpPT0iR3JvdXAiKV0KCm5vcm1fY291bnRzX3N1YiA8LSBub3JtX2NvdW50c19zdWJbb3JkZXIobm9ybV9jb3VudHNfc3ViJFNUOFNJQTFfQjRHQUxOVDEsIGRlY3JlYXNpbmcgPSBUUlVFKSxdCgpwaGVhdG1hcChub3JtX2NvdW50c19zdWIsIGFuZ2xlX2NvbD0iNDUiLCBjZWxsaGVpZ2h0PTEyLCBjbHVzdGVyX3Jvd3MgPSBGQUxTRSwgY2x1c3Rlcl9jb2xzID0gRkFMU0UpCmBgYAoKYGBge3J9CnJtKHRjZ2FTRSkKcm0oY291bnRzX2RkcykKcm0oY291bnRzX3RjZ2EpCnJtKGNvdW50c19kZHNfdGNnYSkKcm0obm9ybV9jb3VudHMpCnJtKG5vcm1fY291bnRzX3N1YikKcm0oZGRzX3RjZ2EpCmdjKCkKYGBgCgojIFNlc3Npb24gaW5mbwoKYGBge3J9CnNlc3Npb25JbmZvKCkKYGBgCgo=
