# Supplementary material for: GD2 Expression in Medulloblastoma and Neuroblastoma for Personalized Immunotherapy: A Matter of Subtype
Source: Cancers (Basel). 2022 Dec 8;14(24):6051. doi: 10.3390/cancers14246051 (PMC9775636; doi:10.3390/cancers14246051)
Supplement: Supplementary file 1 [file cancers-14-06051-s001.zip › Supplementary_figures.pdf]

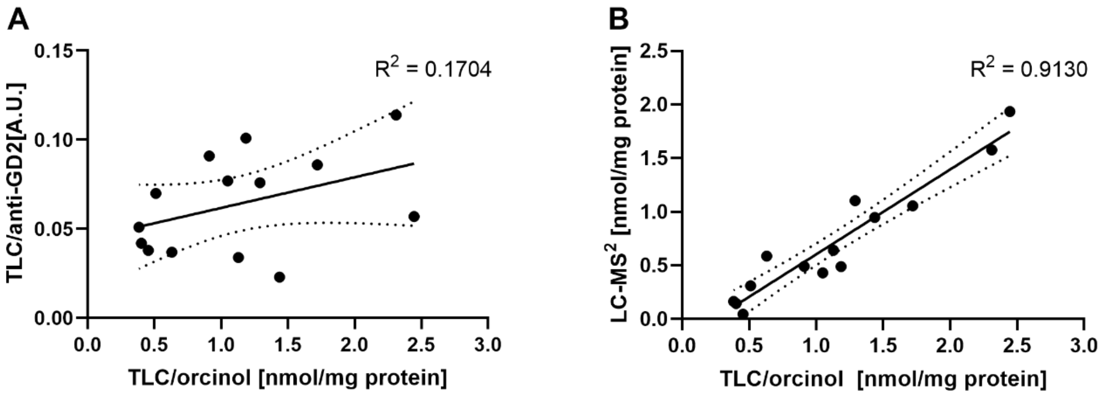

Supplemental figure S1: Correlation of GD2 signals obtained with A) chemical (orcinol reagent) staining on TLC to anti-GD2 immunoverlay staining on TLC or B) chemical (orcinol reagent) staining on TLC to mass spectrometric detection of GD2 eluting from liquid chromatography column (LC-MS^2). Data were entered into GraphPad Prism 9.4.1 software and a simple linear regression was performed showing in addition 95% confidence bands and the corresponding R squared value for the goodness of fit. Note, only the orcinol staining on TLC fits quite well to the quantitative and specific LC-MS^2 measurement and could be used for quantification.

Patient no. 280

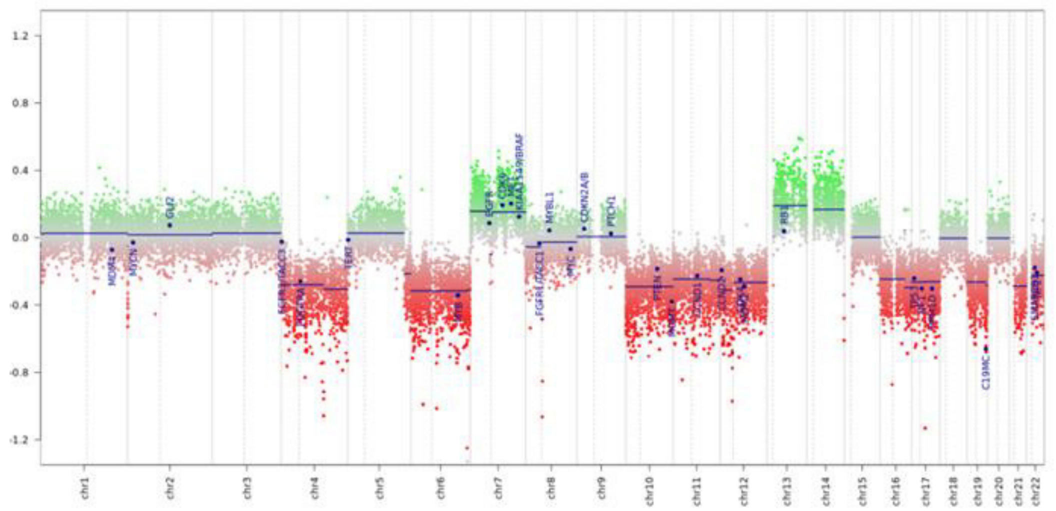

Patient no. 81

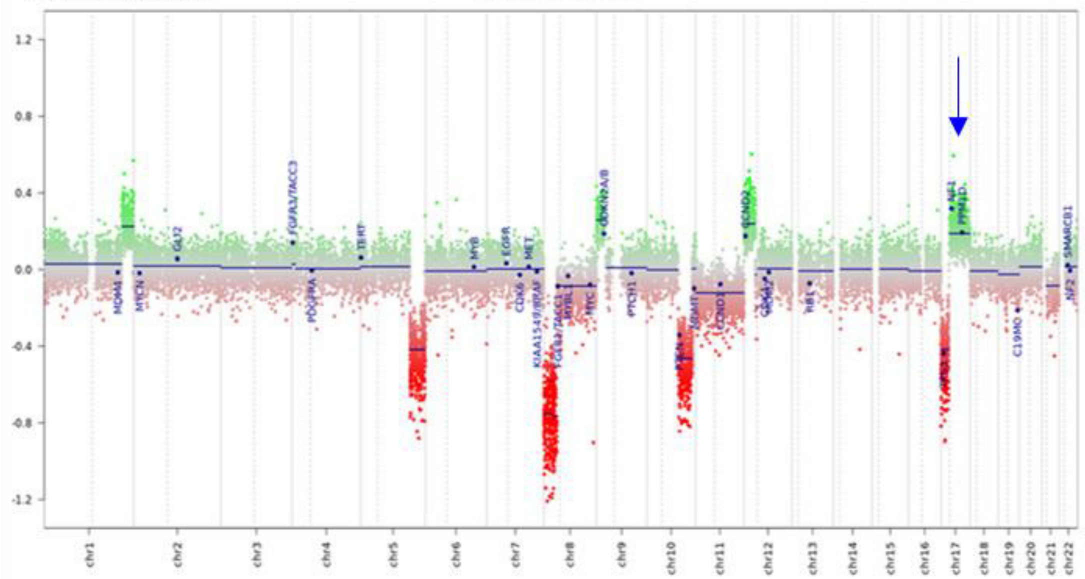

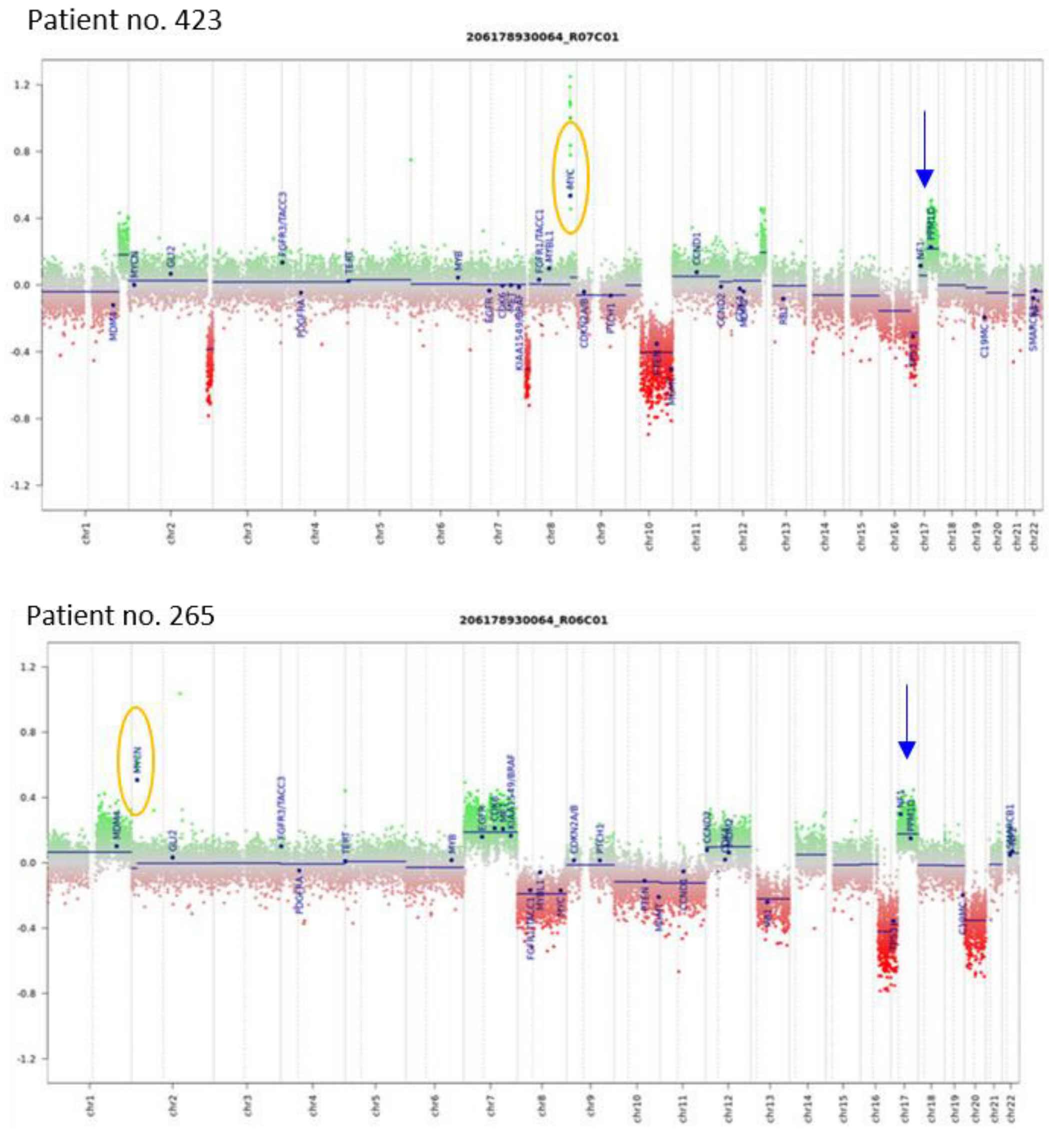

Supplemental figure S2: Copy number variation (CNV) profile of three different MBs group 3 (81, 280, 423) and one MB group 4 (265). Gains/amplifications represent positive (green) and losses represent negative (red) deviations from the baseline. Recurrent gene and chromosomal alterations (e.g., MYC or MYCN amplification (orange circles) and Chr 17q (partial) gain (blue arrows)) are highlighted.

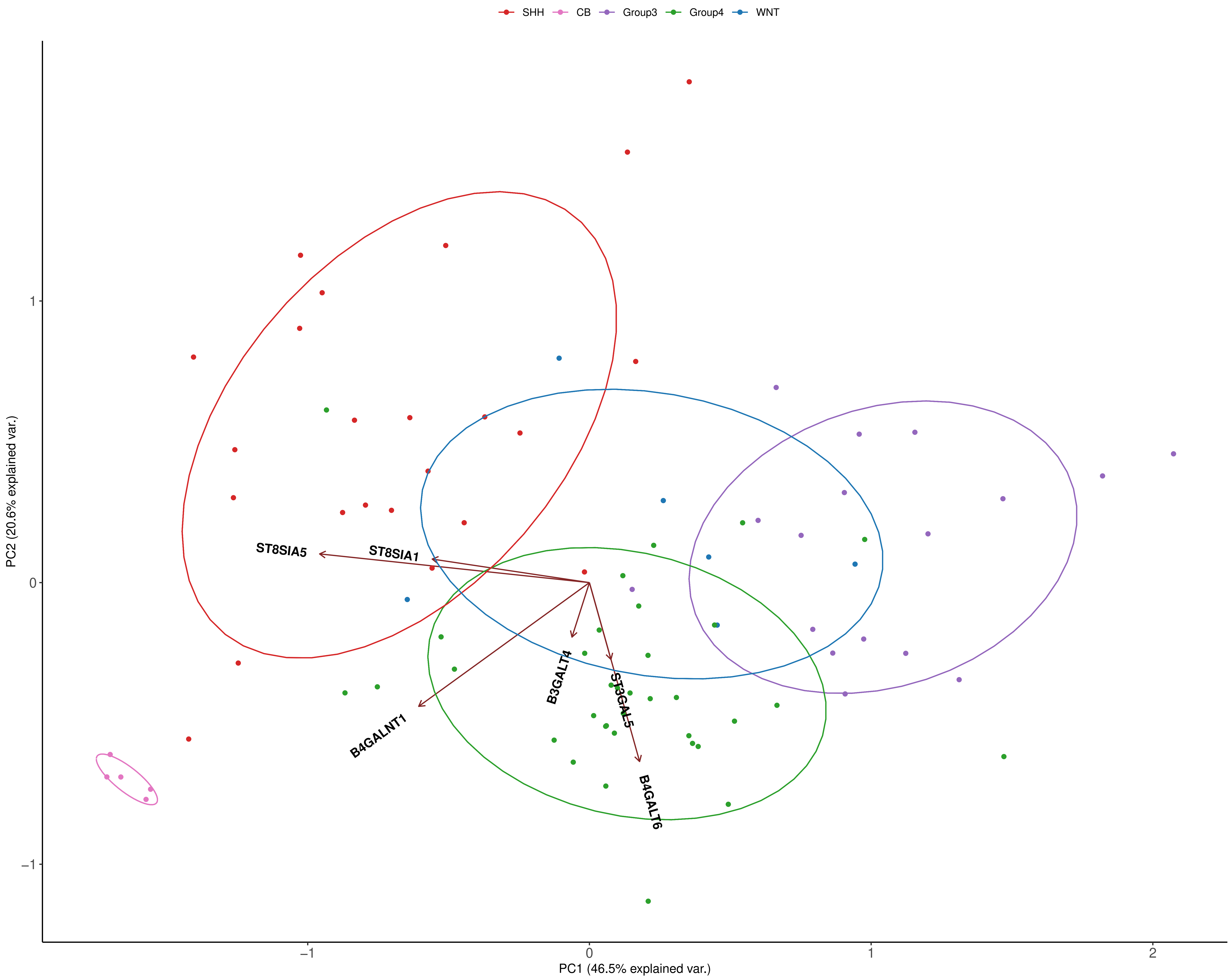

Supplementary figure 3A. High resolution version of figure 4A showing a principal component analysis of gene expression data of six selected genes. Individual samples are represented by colored dots. The color of a dot defines the MB suptype of a sam-ple. The elipses represent the core area of the subtypes by the confidence interval of 68 %. The ar-rows are projections of the original basis vectors (the variables) onto the PC plane.

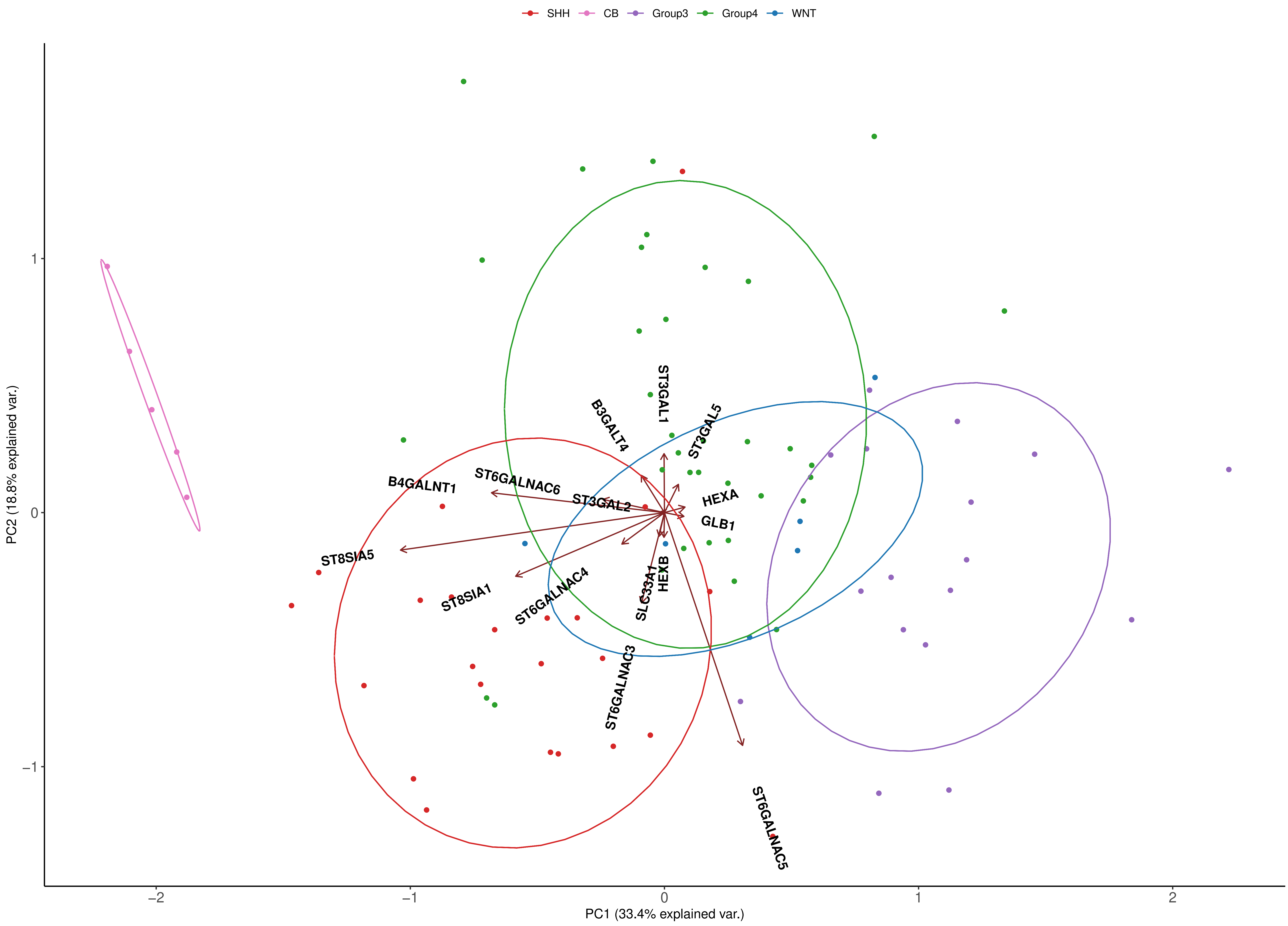

Supplementary figure 3B. High resolution version of figure 4B showing a principal component analysis of genes of the Glycosphingolipid biosynthesis - ganglio series pathway retrieved from KEGG. Individual samples are represented by colored dots. The color of a dot defines the MB suptype of a sam-ple. The elipses represent the core area of the subtypes by the confidence interval of 68 %. The ar-rows are projections of the original basis vectors (the variables) onto the PC plane.
